# Non-Hermitian physics for optical manipulation uncovers inherent instability of large clusters

**DOI:** 10.1038/s41467-021-26732-8

**Published:** 2021-11-15

**Authors:** Xiao Li, Yineng Liu, Zhifang Lin, Jack Ng, C. T. Chan

**Affiliations:** 1 Department of Physics, Southern University of Science and Technology, Shenzhen, Guangdong, 518055 China; 2 Department of Physics, The Hong Kong University of Science and Technology, Hong Kong, China; 3 Institute of Electromagnetics and Acoustics and School of Electronic Science and Engineering, Xiamen University, Xiamen, 361005 China; 4 State Key Laboratory of Surface Physics, Key Laboratory of Micro and Nano Photonic Structures and Department of Physics, Fudan University, Shanghai, China; 5 Collaborative Innovation Center of Advanced Microstructures, Nanjing University, Nanjing, China

**Keywords:** Optical physics, Optical manipulation and tweezers

## Abstract

Intense light traps and binds small particles, offering unique control to the microscopic world. With incoming illumination and radiative losses, optical forces are inherently nonconservative, thus non-Hermitian. Contrary to conventional systems, the operator governing time evolution is real and asymmetric (i.e., non-Hermitian), which inevitably yield complex eigenvalues when driven beyond the exceptional points, where light pumps in energy that eventually “melts” the light-bound structures. Surprisingly, unstable complex eigenvalues are prevalent for clusters with ~10 or more particles, and in the many-particle limit, their presence is inevitable. As such, optical forces alone fail to bind a large cluster. Our conclusion does not contradict with the observation of large optically-bound cluster in a fluid, where the ambient damping can take away the excess energy and restore the stability. The non-Hermitian theory overturns the understanding of optical trapping and binding, and unveils the critical role played by non-Hermiticity and exceptional points, paving the way for large-scale manipulation.

## Abstract (web summary)

Non-conservativeness plays a mysterious role in optical trapping. Applying the non-Hermitian theory, the authors showed that the existence of exceptional points drives the Lorentz force to lose its ability to bind clusters of ~10 or more microparticles, unless remedied by dissipative forces.

## Introduction

Optical trapping (OT) is a process in which optical forces trap particles at the intensity extrema^1–6^. In addition, it has been demonstrated experimentally that quite a large number of particles can be bound by scattering, and this process is called optical binding (OB)^7,8^. Despite decades of research^7–20^, the following question remains unanswered: can OB assemble a large number of particles to create some form of macroscopic “optical matter”? This paper aims to show that this is impossible, even for a modest number of particles (*N* > ~10) in a typical situation. The optically bound clusters found experimentally are actually not bound by light alone. Additional forces, such as dissipative forces arising from viscosity, are indispensable in overcoming instability.

This inevitable instability can be explained by non-Hermitian physics^21–23^. Conventionally, physicists used to focus on Hermitian matrices of conservative closed systems, as they yield real eigenvalues. However, non-Hermitian matrices have recently attracted considerable attention because they can also yield real eigenvalues (for example, in the exact phase of a parity-time symmetric system)^24,25^. In nature, most systems incur losses, and hence, non-Hermitian systems are ubiquitous. The force matrices governing OB stability are actually non-Hermitian because we are dealing with open systems with incoming light and radiative loss^26–31^. More interestingly, they are different from the usual non-Hermitian matrices studied in the exceptional point (EP) literature^32–35^, which typically involve symmetric matrices with complex diagonal terms^36–40^. The force matrices for light-bound clusters are real but asymmetric (thus non-Hermitian) and they possess EPs before which all eigenvalues are real, but turn complex once these EPs are crossed. Thus, a stable OB (stable equilibrium) can only be achieved before reaching the EP. This paper aims to demonstrate that when the number of particles increases in an optically bound cluster, the system will always pass through EPs to yield unstable conjugate pairs of complex eigenvalues, irrespective of details such as particle size, shape, composition, and the illuminating light. We stress that energy is conserved for the entire system composed of light and particles, while non-Hermiticity arises when we consider only the particles’ degrees of freedom (and ignoring the degree of freedom for light), which can exchange energy, momentum, and angular momentum with the light. The “run-away” phenomenon associated with the EP (exponentially growing trajectory when perturbed from the equilibrium) can be suppressed to a certain extent by viscous dissipation^8–11,41–44^.

## Results

### Stability theorem

The system stability is investigated by considering *N* identical spherical particles acted upon solely by optical forces and reaching equilibrium:

1 $$m\frac{{{{{{{\rm{d}}}}}}}^{2}}{{{{{{\rm{d}}}}}}{t}^{2}}\Delta {{{{{\bf{X}}}}}}={{{{{\bf{F}}}}}}(\Delta {{{{{\bf{X}}}}}})\approx \mathop{{{{{\bf{K}}}}}}\limits^{\leftrightarrow}\cdot \Delta {{{{{\bf{X}}}}}},$$

where *m* is the single-particle mass, *t* is the time, $\Delta {{{{{\bf{X}}}}}}=(\Delta {x}_{1},\Delta {y}_{1},\Delta {z}_{1},\cdots ,\Delta {x}_{{{{{{\rm{N}}}}}}},\Delta {y}_{{{{{{\rm{N}}}}}}},\Delta {z}_{{{{{{\rm{N}}}}}}})$ is the displacement from the equilibrium, ${{{{{\bf{F}}}}}}=({F}_{{{{{{{\rm{x}}}}}}}_{1}},{F}_{{{{{{{\rm{y}}}}}}}_{1}},{F}_{{{{{{{\rm{z}}}}}}}_{1}},\cdots ,{F}_{{{{{{{\rm{x}}}}}}}_{{{{{{\rm{N}}}}}}}},{F}_{{{{{{{\rm{y}}}}}}}_{{{{{{\rm{N}}}}}}}},{F}_{{{{{{{\rm{z}}}}}}}_{{{{{{\rm{N}}}}}}}})$ is the optical force, and ${\mathop{{{{{\bf{K}}}}}}\limits^{\leftrightarrow}}_{ij}=\partial {{{{{{\bf{F}}}}}}}_{i}/\partial \Delta {{{{{{\bf{X}}}}}}}_{j}$ is the 3*N* × 3*N* force matrix at the equilibrium^11,41,45^. In general, the forces acting on a collection of *N* spherical lossless particles are conservative if and only if

2 $${{{{{\rm{Work}}}}}}\,{{{{{\rm{done}}}}}}={\oint }_{{{{{{\rm{p}}}}}}}{{{{{\bf{F}}}}}}({{{{{\bf{x}}}}}})\cdot {{{{{\rm{d}}}}}}{{{{{\bf{x}}}}}}={\iint }_{{{{{{\rm{S}}}}}}}\mathop{\sum }\limits_{{\it{i}},{\it{j}}=1}^{{{{{{\rm{3N}}}}}}}\frac{\partial {F}_{{{{{{\rm{j}}}}}}}}{\partial {x}_{{\it{i}}}}{{{{{\rm{d}}}}}}{x}_{{\it{i}}}\wedge {{{{{\rm{d}}}}}}{x}_{{\it{j}}}=0,$$

where **F** and **x** are the vectors (each with 3*N* components) for the optical forces and positions of the *N* particles, *p* is any arbitrary closed path, $S$ is an area bound by *p*, and $\wedge$ denotes the wedge product with ${{{{{\rm{d}}}}}}{x}_{{\it{i}}}\wedge {{{{{\rm{d}}}}}}{x}_{{\it{j}}}=-{{{{{\rm{d}}}}}}{x}_{{\it{j}}}\wedge {{{{{\rm{d}}}}}}{x}_{{\it{i}}}={{{{{\rm{d}}}}}}{x}_{{\it{i}}}{{{{{\rm{d}}}}}}{x}_{{\it{j}}}$. Alternatively, as one can deduce from the above condition, the system is conservative if and only if

3 $$\frac{\partial {F}_{{\it{j}}}}{\partial {x}_{{\it{i}}}}-\frac{\partial {F}_{{\it{i}}}}{\partial {x}_{{\it{j}}}}=0$$

everywhere and for all *i* and *j*. The ${{{{{\bf{F}}}}}}$ and $\mathop{{{{{\bf{K}}}}}}\limits^{\leftrightarrow}$ are numerically evaluated using the rigorous and highly accurate generalized multi-particle Mie theory^46^ and Maxwell stress tensor^47^ (see Methods). Being nonlinear dynamical systems, OT and OB are stable in the Lyapunov sense if and only if their linear approximations in Eq. (1) are stable^48^. In other words, the stability is fully governed by ${K}_{{\it{i}}}$, which represents the eigenvalues of $\mathop{{{{{\bf{K}}}}}}\limits^{\leftrightarrow}$. The general solution to Eq. (1), except at the EP (which is of measure zero), can be expressed as

4 $$\Delta {{{{{\bf{X}}}}}}=\mathop{\sum}\limits_{{\it{i}}=1}^{3N}{\alpha}_{{\it{i}}}\Delta{{{{{{\bf{X}}}}}}}_{0i}{e}^{i{\Omega}_{{\it{i}}}t}=\mathop{\sum }\limits_{{\it{i}}=1}^{3N}{\alpha}_{{\it{i}}}\Delta {{{{{{\bf{X}}}}}}}_{0i}{e}^{i{{{{{\rm{Re}}}}}}({\Omega}_{{\it{i}}})t}{e}^{-{{{{{\rm{Im}}}}}}({\Omega}_{{\it{i}}})t},$$

where ${\alpha }_{i}$ is the complex vibration amplitude for the *i*th mode to be determined by initial conditions, Ω_*i*_ is the generally complex *i*th vibrational frequency, $\Delta {{{{{{\bf{X}}}}}}}_{{{{\rm{0}}}\it{i}}}$ is the *i*th eigenvector of $\mathop{{{{{\bf{K}}}}}}\limits^{\leftrightarrow}$, and ${K}_{{\it{i}}}=-m{{\Omega }_{{\it{i}}}}^{2}$. In brief, a cluster becomes unstable when ${K}_{{\it{i}}}\, > \, 0$ for any *i* (Ω_*i*_ is imaginary) or when ${K}_{{\it{i}}}$ is a complex number for any *i* (Ω_*i*_ is complex). A detailed stability analysis is presented in Supplementary Note 1. This paper focuses on the instability induced by complex ${K}_{{\it{i}}}$, which is related to EPs, and is prevalent in the many-particle limit.

### EPs in OT

To illustrate the basic ideas, we first consider the simplest example of a single-particle OT under a generic incident light, whose generally non-defective real 3 × 3 force matrix can be block-diagonalized into a 2 × 2 real matrix ${\mathop{{{{{\bf{K}}}}}}\limits^{\leftrightarrow}}_{{{{{{\rm{OT}}}}}}}$ and a real scalar^49^. The scalar always corresponds to simple harmonic motion (if stable); thus, we are only concerned with the real matrix. Without loss of generality, upon appropriate rotation, the real matrix ${\mathop{{{{{\bf{K}}}}}}\limits^{\leftrightarrow}}_{{{{{{\rm{OT}}}}}}}$ can be expressed as

5 $${\mathop{{{{{\bf{K}}}}}}\limits^{\leftrightarrow}}_{{{{{{\rm{OT}}}}}}}=\mathop{{{{{\bf{S}}}}}}\limits^{\leftrightarrow}+\mathop{{{{{\bf{A}}}}}}\limits^{\leftrightarrow}=\left[\begin{array}{cc}a+b & 0\\ 0 & a-b\end{array}\right]+\left[\begin{array}{cc}0 & g\\ -g & 0\end{array}\right]$$

where $\mathop{{{{{\bf{S}}}}}}\limits^{\leftrightarrow}=({\mathop{{{{{\bf{K}}}}}}\limits^{\leftrightarrow}}_{{{{{{\rm{OT}}}}}}}+{{\mathop{{{{{\bf{K}}}}}}\limits^{\leftrightarrow}}_{{{{{{\rm{OT}}}}}}}}{\!\,}^{{{{{{\rm{T}}}}}}})/2$ and $\mathop{{{{{\bf{A}}}}}}\limits^{\leftrightarrow}=({\mathop{{{{{\bf{K}}}}}}\limits^{\leftrightarrow}}_{{{{{{\rm{OT}}}}}}}-{{\mathop{{{{{\bf{K}}}}}}\limits^{\leftrightarrow}}_{{{{{{\rm{OT}}}}}}}}{\!\,}^{{{{{{\rm{T}}}}}}})/2$ are, respectively, the conservative symmetric and nonconservative anti-symmetric components, *a* is the average trap stiffness, *b* is half the level spacing between the two trap stiffnesses of $\mathop{{{{{\bf{S}}}}}}\limits^{\leftrightarrow}$, and *g* stems from the nonconservative torque. Equation (5) describes an anisotropic harmonic oscillator associated with $\mathop{{{{{\bf{S}}}}}}\limits^{\leftrightarrow}$ being driven by nonconservative torque associated with $\mathop{{{{{\bf{A}}}}}}\limits^{\leftrightarrow}$. We also note that Eq. (5) can be applied to both 2D and 3D cases, as in the 3D case, we can block diagonalize the $3\times 3$ matrix to obtain Eq. (5).

The eigenvalues of ${\mathop{{{{{\bf{K}}}}}}\limits^{\leftrightarrow}}_{{{{{{\rm{OT}}}}}}}$ are

6 $${K}_{\!\pm }=-m{\Omega }_{\pm }^{2}=\left\{\begin{array}{cc}a\pm 1|\sqrt{{b}^{2}-{g}^{2}}|, & |g| \, < \, |b|,\\ a\pm i|\sqrt{{b}^{2}-{g}^{2}}|, & |g|\, > \, |b|,\end{array}\right.$$

We only consider the case ${{{{\mathrm{Re}}}}}{K}_{\pm }\, < \, 0$, otherwise the system is unstable, independent of the value of *g*. At $g=0$, the system exhibits simple harmonic oscillation about the zero-force position. As evident in Eq. (5), a finite *b* signifies an anisotropy^50,51^ between the two axes, inducing a level spacing in the vibration spectrum. This generates a rotational barrier for the particle, because it prefers to align with one of the axes. When $|g| \, < \, |b|$, the nonconservative torque associated with *g* cannot overcome the barrier *b*. The modes are twisted by *g*, inducing non-orthogonality; otherwise, they are quite similar to harmonic oscillators. For $|g| \, > \, |b|$, a conjugate pair of vibration frequency, Ω_+_ and Ω_−_, emerges, corresponding to stable spiral-in and unstable spiral-out motions, respectively. The point $|g|=|b|$ is the EP, which is the singular point at which the non-Hermitian matrix becomes defective.

**Fig. 1:**
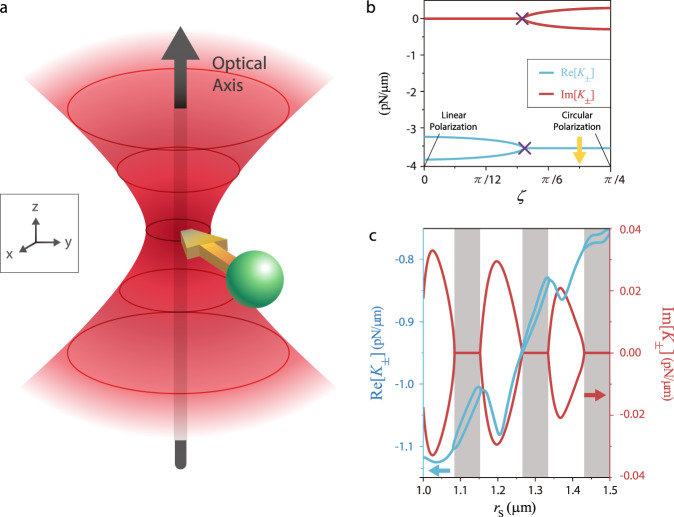
Exceptional point in optical trapping of a single particle. **a** Schematic of the optical trapping of a dielectric particle (${n}_{{{{{{\rm{particle}}}}}}}=1.57$, *r*_S_ = 0.5 μm) by a strongly focused Gaussian beam ($\lambda =1.064$ μm, N.A. = 1.2, filing ratio = 1, power = 1 mW) in water (${n}_{{{{{{\rm{water}}}}}}}=1.33$). **b** ${K}_{{{{{{\rm{i}}}}}}}$ versus the polarization ($\hat{{{{{{\boldsymbol{\varepsilon }}}}}}}=\hat{{{{{{\bf{x}}}}}}}\,\cos \,\zeta +i\hat{{{{{{\bf{y}}}}}}}\,\sin \,\zeta$) of the Gaussian beam, ranging from linear ($\zeta$ = 0) to circular ($\zeta =\pi /4$). The exceptional point of $\mathop{{{{{\bf{K}}}}}}\limits^{\leftrightarrow}$ emerges at $\zeta =2\pi /15$ (marked by purple crosses), beyond which the eigenvalues become complex, which implies instability. **c** The stability also depends on the particle size. ${K}_{{\it{i}}}$ become recursively complex (white region) and real (gray region) as the particle radius is increased. The polarization used here is marked with a yellow arrow in **b**.

Let us consider a dielectric particle trapped by a strongly focused Gaussian beam (standard optical tweezers), as shown in Fig. 1a. Figure 1b plots the eigenvalues ${K}_{\!\pm }$ versus $\zeta$, which defines the beam’s polarization ($\hat{{{{{{\boldsymbol{\varepsilon }}}}}}}=\hat{{{{{{\bf{x}}}}}}}\,\cos \,\zeta +i\hat{{{{{{\bf{y}}}}}}}\,\sin \,\zeta$). The parameter $\zeta$ ranges from 0 (linear polarization) to $\pi /4$ (circular polarization). The conservative part of the force matrix $\mathop{{{{{\bf{S}}}}}}\limits^{\leftrightarrow}$ and the nonconservative part $\mathop{{{{{\bf{A}}}}}}\limits^{\leftrightarrow}$ are, respectively, symmetric and anti-symmetric upon permuting any two indices. Thus, certain symmetries may be compatible with $\mathop{{{{{\bf{S}}}}}}\limits^{\leftrightarrow}$ but not $\mathop{{{{{\bf{A}}}}}}\limits^{\leftrightarrow}$, and vice versa. For linear polarization with $\hat{{{{{{\boldsymbol{\varepsilon }}}}}}}=\hat{{{{{{\bf{x}}}}}}}$ ($\zeta =0$), the *xz*- or *yz*-plane mirror symmetry eliminates $\mathop{{{{{\bf{A}}}}}}\limits^{\leftrightarrow}$ ($g=0$), whereas the asymmetry between *x* and *y* axes allows $b\;\ne\; 0$. The opposite happens for circular polarization with $\hat{{{{{{\boldsymbol{\varepsilon }}}}}}}=(\hat{{{{{{\bf{x}}}}}}}+i\hat{{{{{{\bf{y}}}}}}})/\sqrt{2}$ ($\zeta =\pi /4$). The cylindrical symmetry of the beam enforces $b=0$, whereas the finite angular momentum of light gives $g\,\ne\, 0$^50^. Consequently, an EP where $|b|=|g|$ must exist for an intermediate $\zeta$ between 0 ($|b|\,\ne\, 0,|g|=0$) and $\pi /4$ ($|b|=0,|g|\,\ne\, 0$). As shown in Fig. 1b, as the value of $\zeta$ increases, the two originally real eigenvalues (light blue lines) coalesce at the EP marked by purple crosses, whereas the imaginary part bifurcates into a conjugate pair. Consequently, once passed through the EP, ${{{{{\rm{Im}}}}}}({\Omega}_{{\it{-}}})\, < \, 0$, rendering the system unstable, as shown in Eq. (4). The instability originates from the nonconservative torque (associated with *g*), which exceeds the half level spacing (*b*), as shown in Eq. (6). This overturns the intuitive but inaccurate common understanding that instability is always due to the nonconservative force overcoming the conservative one, or the conservative force itself is unstable. It is somewhat surprising that it is the level spacing characterized by *b* that plays the role in stability. Even when both trap stiffnesses are very large (i.e., $a\to -\infty$), the complex instability still emerges whenever |*g*| > |*b*|. Figure 1c plots ${K}_{\!\pm }$ against the particle radius at an elliptical polarization marked by the yellow arrow in Fig. 1b ($\zeta ={37.5}^{\circ }$). Here, *b* and *g* vary with the radius, creating alternating intervals of real (gray region) and complex (white region) eigenvalues. When *g* exceeds *b*, the particle orbits around the trap with increasing speed, and eventually being torn away by the centrifugal force^45^.

### EPs in OB

Meanwhile, EPs are also found in OB. It was experimentally demonstrated by Burns et al.^7,8^ that analogous to the binding of matter by electrons, light can bind microparticles into a form of soft condensed matter, known as “optical matter”. We will see that, unlike electrons, photons cannot bind many particles because the laser power is limited, and more importantly, the electronic Hamiltonian is Hermitian, but its counterpart in OB, $\mathop{{{{{\bf{K}}}}}}\limits^{\leftrightarrow}$, is manifestly non-Hermitian.

**Fig. 2:**
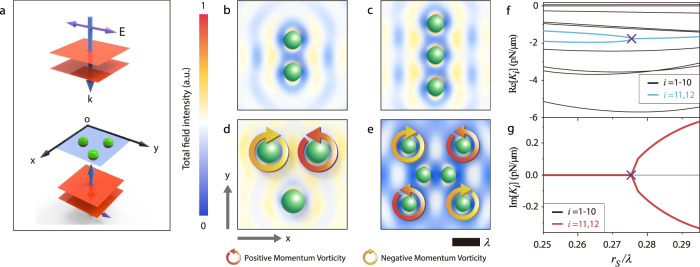
Exceptional points in optical binding. **a** Schematic of a pair of counter-propagating plane waves that are applied to confine particles on the *xy* plane. Despite the incident light intensity is uniform over the *xy* plane, the scattering of light among the spheres modifies the spatial light distribution, as shown in **b**–**e**, which induces binding forces. The colored contour plots in **b**–**e** represent the normalized total field intensity for the spheres with **b** ${r}_{{{{{{\rm{S}}}}}}}=0.31\lambda$ and ${n}_{{{{{{\rm{r}}}}}}}=1.2$, **c** ${r}_{{{{{{\rm{S}}}}}}}=0.31\lambda$ and ${n}_{{{{{{\rm{r}}}}}}}=1.2$, **d** ${r}_{{{{{{\rm{S}}}}}}}=0.41\lambda$ and ${n}_{{{{{{\rm{r}}}}}}}=1.2$, and **e** ${r}_{{{{{{\rm{S}}}}}}}=0.27\lambda$ and ${n}_{{{{{{\rm{r}}}}}}}=1.51$. All figures are drawn to scale. Red (yellow) circles denote positive (negative) momentum vorticity *C* (see main text) near each sphere. **f**–**g** plot the real (**f**) and imaginary (**g**) components of the eigenvalues for the force matrix of **e** against the particle radius. The exceptional point is marked by purple crosses.

As shown in Fig. 2b–e, particle clusters are trapped and bounded on the *xy*-plane by the incident field depicted in Fig. 2a. The mirror symmetries about the *xz*- and *yz*-planes decouple the *z* motion from the *x* and *y* motion; thus, we focus on the *x* and *y* motion. The incident wave possesses neither energy flow (so scattering force vanishes) nor intensity gradient (so gradient force vanishes) along the *x* and *y* directions^52^; and hence any transverse force that occurs is exclusively owing to scattering, which modifies the spatial light distribution to induce OB. As evident in Fig. 2b–e, the total field is drastically different from the uniform incident field.

Let us consider the “momentum vorticity”, defined as ${{{{{\rm{C}}}}}}={\oint }_{{{\rm{particle}}}\ {{\rm{circumference}}}}\,{{{{{\bf{p}}}}}}\cdot d{{{{{\bf{r}}}}}}$, where ${{{{{\bf{p}}}}}}$ represents the photon momentum density. In Fig. 2d–e, the red and yellow circles indicate $C\, > \, 0$ and $C\, < \, 0$, respectively. $C\,\ne\, 0$ implies the field tends to rotate the particle, favoring the occurrence of complex modes (CMs). Particles not marked by a circle have $C=0$, which is enforced by the mirror symmetries, but that does not exclude CMs. Consider the component ${\mathop{{{{{\bf{A}}}}}}\limits^{\leftrightarrow}}_{{{{{{\rm{\mu }}}}}}{{{{{\rm{\nu }}}}}}}=1/2[\partial {F}_{{{{{{\rm{\mu }}}}}}}/\partial {x}_{{{{{{\rm{\nu }}}}}}}-\partial {F}_{{{{{{\rm{\nu }}}}}}}/\partial {x}_{{{{{{\rm{\mu }}}}}}}]$, it can couple the coordinates $\mu$ and $\nu$ in a non-Hermitian manner, thus inducing CMs if it is sufficiently large, even when the “momentum vorticity” is absent. Here, $\mu$ and $\nu$ each label an arbitrary cartesian coordinate of an arbitrary particle. This is similar to how $\partial {F}_{{{{{{\rm{x}}}}}}}/\partial y-\partial {F}_{{{{{{\rm{y}}}}}}}/\partial x=2g$ couples *x* and *y* in single-particle OT.

The configuration shown in Fig. 2b is a chain of two spheres, which possesses four modes in *xy*-plane, and the corresponding force matrix is

7 $$\mathop{{{{{\bf{K}}}}}}\limits^{\leftrightarrow}=\left[\begin{array}{cccc}0.027 & 0 & -0.027 & 0\\ 0 & -0.580 & 0 & 0.580\\ -0.027 & 0 & 0.027 & 0\\ 0 & 0.580 & 0 & -0.580\end{array}\right]\frac{{{{{{\rm{pN}}}}}}}{{{{{{\rm{\mu }}}}}}{{{{{\rm{m}}}}}}}.$$

The mirror symmetries about the *xz*- and *yz*-planes and the translational invariance along *x* and *y* directions make the force matrix completely symmetric (i.e., Hermitian) and thus the eigenvalues must be real. However, increasing the number of particles (e.g., three spheres in Fig. 2c) will break the Hermiticity. More particles imply more degrees of freedom but the number of available spatial symmetries does not increase indefinitely. This breaks the Hermiticity because the symmetry is insufficient to make the force matrix fully symmetric. For example, despite it sharing the same symmetry as the two spheres case, the force matrix for the three-sphere chain (Fig. 2c) is asymmetric:

8 $$\mathop{{{\bf{K}}}}\limits^{\leftrightarrow}=\left[\begin{array}{cccccc}0.010 & 0 & -0.006 & 0 & -0.004 & 0\\ 0 & -0.159 & 0 & 0.140 & 0 & 0.019\\ -0.007 & 0 & 0.015 & 0 & -0.007 & 0\\ 0 & 0.139 & 0 & -0.278 & 0 & 0.139\\ -0.004 & 0 & -0.006 & 0 & 0.010 & 0\\ 0 & 0.019 & 0 & 0.140 & 0 & -0.159\end{array}\right]\frac{{{{\rm{pN}}}}}{{{{\rm{\mu }}}}{{{\rm{m}}}}}.$$

In addition to the number of particles, symmetry-breaking also increases the non-Hermiticity. The triangular structure comprising of 3 particles in Fig. 2d exhibits a single mirror symmetry on the *yz*-plane (less than the two mirror symmetries for the chain in Fig. 2c), which makes the force matrix possess even more asymmetric components:

9 $$\mathop{{{\bf{K}}}}\limits^{\leftrightarrow}=\left[\begin{array}{cccc}0.027 & 0 & -0.027 & 0\\ 0 & -0.580 & 0 & 0.580\\ -0.027 & 0 & 0.027 & 0\\ 0 & 0.580 & 0 & -0.580\end{array}\right]\frac{{{{\rm{pN}}}}}{{{{\rm{\mu }}}}{{{\rm{m}}}}}.$$

In Eqs. (8) and (9), the ratio of the asymmetric off-diagonal pairs are, respectively, 4:15, and 12:15, i.e., the one with fewer spatial symmetries clearly has more asymmetric matrix elements. Detailed discussion and information about the force matrices (Eqs. (7–9)) can be found in Supplementary Note 2. We note that, for the triangular geometry, EPs can already exist although they are rare (see Supplementary Note 2).

The cluster of six particles in Fig. 2e exhibits mirror symmetries about the *xz*- and *yz*-planes. However, given the 2*N* = 12 transverse degrees of freedom, the symmetries cannot make the force matrix symmetric. Consequently, EPs can emerge easily. For the geometry shown in Fig. 2e, ${{{{\mathrm{Re}}}}}({K}_{{{{{{\rm{i}}}}}}})$ and ${{\mbox{Im}}}({K}_{{{{{{\rm{i}}}}}}})$ are plotted against the particle radius in Fig. 2f, g, respectively. At each radius, the configuration is relaxed to find the equilibrium positions for the particles. Ten modes are real (black) in this parameter range, whereas the remaining two (light blue) are initially real but become complex after reaching the EP, which is marked by purple crosses in the figure. After crossing the EP, the real parts (light blue) merge, whereas the originally zero imaginary part (red) splits into an equal but opposite pair of CMs. In Supplementary Note 2, other examples of optically bound structures with EP are presented, all of them corroborate the analysis.

### Prevalence of CMs in OB systems

**Fig. 3:**
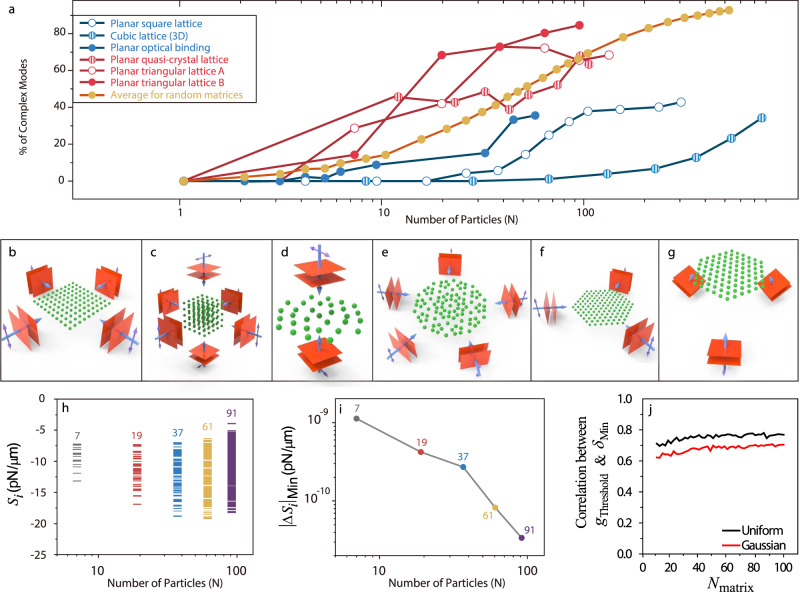
Prevalence of complex modes with increasing particles. **a** Percentage of complex modes (CMs) increases with particle number for all considered geometries. **b** Planar square lattice (${r}_{{{{{{\rm{S}}}}}}}=0.2\lambda$, ${n}_{{{{{{\rm{r}}}}}}}=1.2$) bound by four in-plane plane waves. **c** Cubic lattice (3D) (${r}_{{{{{{\rm{S}}}}}}}=0.2\lambda$, ${n}_{{{{{{\rm{r}}}}}}}=1.2$) bound by six plane waves. **d** Planar OB confined by two counter-propagating plane waves. **e** Planar quasi-crystal lattice (${r}_{{{{{{\rm{S}}}}}}}=0.2\lambda$, ${n}_{{{{{{\rm{r}}}}}}}=1.1$) bound by five in-plane plane waves. **f** Planar triangular lattice A (${r}_{{{{{{\rm{S}}}}}}}=0.2\lambda$, ${n}_{{{{{{\rm{r}}}}}}}=1.1$) bound by three in-plane plane waves. **g** Planar triangular lattice B (${r}_{{{{{{\rm{S}}}}}}}=1.7{{{{{\rm{\mu }}}}}}{{{{{\rm{m}}}}}}$, $\lambda =0.5145{{{{{\rm{\mu }}}}}}{{{{{\rm{m}}}}}}$, ${n}_{{{{{{\rm{r}}}}}}}=1.57/1.33$) bound by three nearly *x*-polarized, *z*-propagating plane waves with the angle between their *k*-vectors being ~2°. This configuration was also considered in ref. ^8^. The details regarding the incident waves and the particle arrangement for **b**–**g** are presented in Supplementary Note 3. **h** The distribution of the eigenvalues of $\overleftrightarrow{{{{{{\bf{S}}}}}}}$ for the geometry in **g**. The density of eigenvalues within a specific spectral interval increases with *N*. **i** The minimum level spacing (${|\Delta {S}_{{{{{{\rm{i}}}}}}}|}_{{{{{{\rm{Min}}}}}}}$) decreases with *N*. **j** Correlation between ${g}_{{{{{{\rm{Threshold}}}}}}}$ and ${\delta }_{{{{{{\rm{Min}}}}}}}$ for each matrix size, constructed from uniform or Gaussian random numbers.

Figure 3a shows the percentage of CMs versus cluster size. Each colored line represents a class of geometry depicted in Fig. 3b–g. The CM percentages increase with *N* and become dominant for a large value of *N*. The red (blue) lines correspond to an incident non-standing (standing) wave, which does (does not) directly exert a nonconservative force on the individual particles^52,53^. The results show that it is more likely for the non-standing wave to induce complex eigenvalues and hence the red lines rise faster than the blue lines. For a cluster bound by a non-standing wave, the first pair of CMs emerges at $N \, < \, 10$ and most modes are complex when $N \, > \, 50$. All clusters with $N \, > \, 70$ possess CMs, demonstrating the necessity to account for the CM physics even for moderately sized clusters. The line with red solid circles in Fig. 3a corresponds to the experimental configuration in ref. ^8^, where CMs exist for all calculated cluster sizes, ranging from *N* = 7–91. The non-Hermitian physics analysis implies that the results referred to in ref. ^8^ requires an alternative interpretation: the optical forces alone are insufficient to produce optical crystallization^8,54^, and additional forces, such as viscosity, are essential.

Figure 3h shows the mode distribution for the eigenvalues ${S}_{i}$ of $\mathop{{{{{\bf{S}}}}}}\limits^{\leftrightarrow}=(\mathop{{{{{\bf{K}}}}}}\limits^{\leftrightarrow}+{\mathop{{{{{\bf{K}}}}}}\limits^{\leftrightarrow}}^{{{{{{\rm{T}}}}}}})/2$ for the configuration shown in Fig. 3g. As *N* ranges from 7 to 91, the “bandwidth” of the eigenvalues doubles, whereas the number of eigenmodes increases by 13 times. This implies an ~6.5-fold increase in the average “density of eigenvalues” within a specific spectral interval and a corresponding decrease in the gap separating adjacent eigenvalues. The decrease of the gaps between eigenvalues with increasing *N* is universal, independent of details. The conservative force $\mathop{{{{{\bf{S}}}}}}\limits^{\leftrightarrow}$ is induced by the external light source with a finite intensity, thus ${S}_{{{{{{\rm{i}}}}}}}$ is bounded by a lower bound ${S}_{{{{{{\rm{lower}}}}}}}$ and an upper bound ${S}_{{{{{{\rm{upper}}}}}}}$. Then, for *N* particles, there will be 3*N* modes within a finite and fixed interval $[{S}_{{{{{{\rm{lower}}}}}}},{S}_{{{{{{\rm{upper}}}}}}}]$ independent of *N*. Evidently, the average level spacing has to diminish to zero as $N\to \infty$ (Fig. 3i). The inherent non-Hermiticity will have an increasingly high probability to coalesce some of the modes as the level spacings get smaller and smaller. As a consequence, the encounter with an EP becomes inevitable in the large *N* limit. The emergence of complex eigenvalues in the many-particle limit is in fact a mathematical consequence of dealing with large asymmetric real matrices. It is known that the eigenvalues for a sufficiently large random asymmetric matrix are complex, even for the tiniest asymmetry^55^. We generated 10,000 random matrices (see Methods for definition) for each matrix size (*N*_matrix_), with *N*_matrix_ ranging from 10 to 100 in steps of 2. We define ${g}_{{{{{{\rm{Threshold}}}}}}}$ as the minimum asymmetry in the matrix required to generate the first pair of CMs (see Methods) and ${\delta }_{{{{{{\rm{Min}}}}}}}$ as the minimum level spacing. For any *N*_matrix,_ the correlation (shown in Fig. 3j) between ${g}_{{{{{{\rm{Threshold}}}}}}}$ and ${\delta }_{{{{{{\rm{Min}}}}}}}$ are ~0.75 and ~0.65 for uniform and Gaussian random numbers, respectively. Clearly the asymmetry (${g}_{{{{{{\rm{Threshold}}}}}}}$) required to induce CMs is smaller when the minimum level spacing (${\delta }_{{{{{{\rm{Min}}}}}}}$) is smaller. The minimum level spacing vanishes in the large *N*_matrix_ limit, so does ${g}_{{{{{{\rm{Threshold}}}}}}}$, indicating even a tiny asymmetry is sufficient to generate CMs when the matrix size is large. The average percentage of CMs for the random matrix filled with uniform random numbers is also plotted in Fig. 3a. The trend is similar to the optically bound clusters. These results are in good agreement with our analysis.

### Stability phase diagrams

**Fig. 4:**
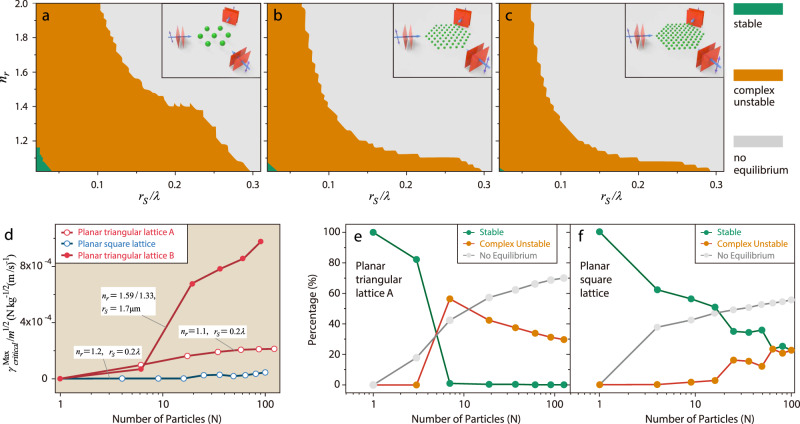
Stability phases for the optically bound triangular lattices. Gray areas denote the absence of equilibrium (zero-force configuration). The green areas denote stability regions where light alone stabilizes the cluster (i.e., optical binding). Orange areas denote the region where damping is required to stabilize the cluster (opto-hydrodynamic binding). **a** *N* = 7, **b** *N* = 61, **c** *N* = 91. **d** Minimum damping coefficient required to stabilize the cluster versus *N*. A larger damping value is required at a larger *N*. **e**–**f** Percentages of the phase space area occupied by OB, opto-hydrodynamic binding, and no-equilibrium phase, with **e** representing the triangular lattice and **f** the square lattice. The lines linking the points are guides to the eyes only.

Figure 4a–c shows the stability phase diagrams for the triangular lattices of particles trapped by three in-plane plane waves (see insets). It is now well-accepted in the literature that optical force alone can bind particles into a stable entity. Although it is true for a small cluster of particles with a small refractive index, OB is stable as shown in Fig. 4 only in a very small green domain in the phase diagram, and the stable domain diminishes with increasing *N*. Evidently, the interpretation of OB requires refinement, especially when the scattering is strong such that the nonconservative force becomes prominent. In the gray domains, equilibrium configurations cannot be found, indicating the absence of optical crystallization (trapping a large number of particles at the intensity extrema of an optical lattice)^56^. This is a consequence of having an OB force induced by light scattering being greater than the OT force induced by the incident field. In the orange domains, zero-force positions can be found but those are unstable equilibria because the clusters possess CMs. Such systems are unstable when acted upon by optical force alone but can be stabilized with sufficient damping (when the damping coefficient $\gamma \, > \, {\gamma }_{{{{{{\rm{critical}}}}}}}^{{{{{{\rm{i}}}}}}}=\sqrt{m}|{{{\mbox{Im}}}}({K}_{{{{{{\rm{i}}}}}}})|/\sqrt{|{{{{{\mathrm{Re}}}}}}({K}_{{{{{{\rm{i}}}}}}})|}$ for all modes^11^). When the particles are submerged in a fluid, the hydrodynamic damping^57,58^ provides the dissipation needed to damp out unstable CMs. Similar behaviors should be qualitatively expected for all types of dissipative forces added to OB, because the key to stabilize OB lies in dissipating the energy injected by light. In the present study, dynamic simulations were performed on some particle clusters experiencing optical force and hydrodynamic forces. The outcome was qualitatively similar to the case where only optical forces and damping were considered (see Supplementary Note 4 and Supplementary Movies 1–2).

As evident in Fig. 4d, the required damping for stabilization generally increases with *N*. Figure 4e, f shows the fraction of area covered by stable, complex unstable, and no-equilibrium phases. As *N* increases, the no-equilibrium phases become increasingly dominant, whereas the stable domains described in ref. ^8^ contract. The complex unstable domains first expand by replacing the stable domain and then shrink as they are replaced by the non-equilibrium domains. Optical crystallization cannot be achieved in the gray domains even with damping. Thus, the particles do not always follow the predefined incident interference pattern^56^.

## Discussion

Contrary to the insightful proposal of OB put forth previously^7,8^, the optical force gradually loses its ability to stabilize a cluster with an increasing number of particles, because the nonconservative force is allowed to continuously “pump” energy into the system, which finally “melts” the system. Nevertheless, the stability of such a system can be retained by introducing sufficient damping, which prevents energy accumulation. The fact that dissipation in water damps out the non-Hermitian instability does not mean non-Hermiticity has no consequence. We take single-particle trapping as an example, Svak, V. et al.^44^ have shown the equipartition theorem is broken in the presence of nonconservative forces. This is in fact also a consequence of the system’s non-Hermiticity. Our work offers a new perspective on OT and OB. In addition, our theory is applicable to other non-Hermitian systems as well, including, but not limited to, acoustic trapping and binding.

Vacuum OT^59^ and OB have emerged as a hot and important topic, partly because of the possibility to realize quantum entanglement of macroscopic objects^60,61^. In fact, vacuum OB has been experimentally realized^19,20^. In these experiments, since only a few particles are involved, the non-Hermitian effect is negligible. Nevertheless, in Supplementary Note 5, we show that in agreement with the conclusion made here, when the number of particles increases, or when the certain system symmetry is broken, the non-Hermiticity should be taken into account for a complete description. In vacuum, optical crystallization and OB are generally unstable in the many-particle limit. In water, sufficient damping can remedy the trapping. As such, OT and OB in water, while primarily due to the Lorentz force, must be assisted by damping, and are better described as “opto-hydrodynamic trapping and binding”. All these are consequences of the inherent non-Hermitian feature of an open system that possesses EPs.

We note that there are very special configurations in which an optical cluster can grow infinitely big. For example, an infinite one-dimensional periodic chain of particles (with no boundaries) possesses a Hermitian $\mathop{{{{{\bf{K}}}}}}\limits^{\leftrightarrow}$, and thus, is not subjected to complex instability even when $N\to \infty$. However, under the many-particle limit, as the level spacing approaches zero, the smallest amount of symmetry breaking will introduce nonconservative forces that will destabilize the system. In other words, the protecting symmetry must be “perfect”. More specifically, all particles are required to be perfectly identical, all counter-propagating waves should have the same amplitude and matching phases, etc. In practice, such stability is fragile, as constructing an infinite periodic lattice of identical particles is basically impossible to realize experimentally. We remark that if we replace ideal spherical particles that have no internal degree of freedom with a non-spherical particle that has some structural degrees of freedom, the instability will be even more likely to occur because there are more degrees of freedom in the system at the same number of particles.

Finally, our work has an impact beyond OT and can be applied to study any large non-Hermitian systems, which can be in optics, mechanics, acoustics, etc. Some examples, which warrant further investigations, are listed here. (1) Whenever we use a wave to trap particles, such as in acoustic trapping^62,63^ or OT, energy can be exchanged between waves and particles, rendering the particle system alone nonconservative. Such systems would possess non-Hermitian $\mathop{{{{{\bf{K}}}}}}\limits^{\leftrightarrow}$. In fact, the transition from sufficiently damped stable complex modes to insufficiently damped unstable complex modes were observed in acoustic trapping for a single trapped particle^64^, although its non-Hermitian characteristics were not recognized. (2) Anti-symmetric real matrix is seldom found in other non-Hermitian systems, which are usually represented by symmetric matrices (reciprocal coupling) with complex diagonal terms (indicating explicit gain/loss). It extends the realm of non-Hermitian physics to an under-explored regime that cannot be easily realized otherwise. (3) The emergence of complex modes with increasing matrix size is a mathematical consequence for non-Hermitian systems. As such, the emergence of complex eigenvalues for large matrices is a universal phenomenon that can also be expected in another large non-Hermitian system.

## Methods

### Computing electromagnetic fields for a collection of spherical particles

The Maxwell equations governing the behavior of light are solved by the multi-particle generalization of the Mie theory^46^. In short, the incident and scattered waves of each sphere are expanded in a series of vector spherical wavefunctions. Applying the vector translation–addition theorem and the standard electromagnetic boundary conditions, the expansion coefficients can be determined by solving a linear system of equations. Such a semi-analytical approach is highly efficient and accurate.

### Modeling the strongly focused Gaussian beam

A strongly focused Gaussian beam is modeled by the vector generalization of the Debye integral^41^. The lens is much larger than the wavelength, and thus, geometrical optics can be applied to solve the focusing problem. Then, using the Debye integral, the full-wave electromagnetic field in the focus region can be determined by connecting it to the geometrical optics solution. Such an approach is known to yield accurate results that can be directly compared with the experimental results.

### Computing the optical force

The time-averaged optical force, referred to as optical force in this paper, can be calculated using the surface integral of the time-averaged Maxwell stress tensor^47^:

10 $${{{{{\bf{F}}}}}}={{\oint }_{{{{{{\rm{S}}}}}}}\langle \mathop{{{{{\bf{T}}}}}}\limits^{\leftrightarrow}\rangle }_{{{{{{\rm{t}}}}}}}\cdot {{{{{\rm{d}}}}}}{{{{{\bf{a}}}}}},$$

where ${\langle \mathop{{{{{\bf{T}}}}}}\limits^{\leftrightarrow}\rangle }_{{{{{{\rm{t}}}}}}}$ is the time-averaged Maxwell stress tensor, and the electromagnetic fields required to evaluate this tensor are obtained by the Mie theory. This approach is highly accurate.

### Searching for equilibrium positions

The equilibrium configurations are identified by performing dynamic simulations to propagate the particle motion forward in time inside a fictitious medium with damping using an efficient integrator. The simulation stops once we find an equilibrium where the forces acting on all particles become zero.

### Evaluating the force matrix and identifying the EPs

Once equilibrium is reached, we numerically evaluate the force matrix, ${\mathop{{{{{\bf{K}}}}}}\limits^{\leftrightarrow}}_{{{{{{\rm{ij}}}}}}}=\partial {{{{{{\bf{F}}}}}}}_{{{{{{\rm{i}}}}}}}/\partial \Delta {{{{{{\bf{x}}}}}}}_{{{{{{\rm{j}}}}}}}$, by using the finite difference method. If $\mathop{{{{{\bf{K}}}}}}\limits^{\leftrightarrow}$ is found to be defective at a certain value of a parameter, it is called an EP.

### Definition of random matrix

Any matrix $\mathop{{{{{\bf{K}}}}}}\limits^{\leftrightarrow}$ can be split into a symmetric part $\mathop{{{{{\bf{S}}}}}}\limits^{\leftrightarrow}=(\mathop{{{{{\bf{K}}}}}}\limits^{\leftrightarrow}+{\mathop{{{{{\bf{K}}}}}}\limits^{\leftrightarrow}}{\,\!}^{{{{{{\rm{T}}}}}}})/2$ and an anti-symmetric part $\mathop{{{{{\bf{A}}}}}}\limits^{\leftrightarrow}=({\mathop{{{{{\bf{K}}}}}}\limits^{\leftrightarrow}-\mathop{{{{{\bf{K}}}}}}\limits^{\leftrightarrow}}{\,\!}^{{{{{{\rm{T}}}}}}})/2$. By applying a similarity transformation $\mathop{{{{{\bf{U}}}}}}\limits^{\leftrightarrow}$ that diagonalizes $\mathop{{{{{\bf{S}}}}}}\limits^{\leftrightarrow}$, the original matrix becomes

11 $${\mathop{{{{{\bf{U}}}}}}\limits^{\leftrightarrow}\mathop{{{{{\bf{K}}}}}}\limits^{\leftrightarrow}\mathop{{{{{\bf{U}}}}}}\limits^{\leftrightarrow}}{\,\!}^{{{{{{\rm{T}}}}}}}={\mathop{{{{{\bf{U}}}}}}\limits^{\leftrightarrow}\mathop{{{{{\bf{S}}}}}}\limits^{\leftrightarrow}\mathop{{{{{\bf{U}}}}}}\limits^{\leftrightarrow}}{\!\,}^{{{{{{\rm{T}}}}}}}+{\mathop{{{{{\bf{U}}}}}}\limits^{\leftrightarrow}\mathop{{{{{\bf{A}}}}}}\limits^{\leftrightarrow}\mathop{{{{{\bf{U}}}}}}\limits^{\leftrightarrow}}{\!\,}^{{{{{{\rm{T}}}}}}}=\mathop{{{{{\bf{S}}}}}}\limits^{\leftrightarrow}{\,\!}^{\prime} +\mathop{{{{{\bf{A}}}}}}\limits^{\leftrightarrow}{\,\!}^{\prime},$$

where $\mathop{{{{{\bf{S}}}}}}\limits^{\leftrightarrow}{\,\!}^{\prime}$ is a diagonal matrix and $\mathop{{{{{\bf{A}}}}}}\limits^{\leftrightarrow}{\,\!}^{\prime}$ is an anti-symmetric matrix. We generate random matrices $\mathop{{{{{\bf{S}}}}}}\limits^{\leftrightarrow}{\,\!}^{\prime}$ and $\mathop{{{{{\bf{A}}}}}}\limits^{\leftrightarrow}{\,\!}^{\prime}$ that have similar forms to study their threshold behavior. We define

12 $${\overleftrightarrow{{{{{{\bf{M}}}}}}}}_{N\times N}={\mathop{{{{{\bf{S}}}}}}\limits^{\leftrightarrow}}_{{{{{{\rm{random}}}}}}}+{g}_{{{{{{\rm{asym}}}}}}}{\overleftrightarrow{{{{{{\rm{A}}}}}}}}_{{{{{{\rm{random}}}}}}},$$

where ${\mathop{{{{{\bf{S}}}}}}\limits^{\leftrightarrow}}_{{{{{{\rm{random}}}}}}}$ is a random diagonal matrix with elements ${a}_{{{{{{\rm{i}}}}}}}$ uniformly distributed between −1 and 0, whereas ${\mathop{{{{{\bf{A}}}}}}\limits^{\leftrightarrow}}_{{{{{{\rm{random}}}}}}}$ is an anti-symmetric matrix with random elements ${g}_{{{{{{\rm{jk}}}}}}}=-{g}_{{{{{{\rm{kj}}}}}}}$ uniformly distributed between −1 and 1. Here ${g}_{{{{{{\rm{asym}}}}}}}$ tunes the strength of the anti-symmetric non-Hermitian term. The minimum level spacing ${\delta }_{{{{{{\rm{Min}}}}}}}$ is the minimum value of $|{a}_{{{{{{\rm{i}}}}}}}-{a}_{{{{{{\rm{j}}}}}}}|$ for any pair of *i* and *j* where $i\,\ne\, j$. For each random matrix, we calculate ${g}_{{{{{{\rm{Threshold}}}}}}}$, the minimum ${g}_{{{{{{\rm{asym}}}}}}}$ required to generate the first pair of CMs, by using bisection. Random numbers ${a}_{{{{{{\rm{i}}}}}}}$ and ${g}_{{{{{{\rm{jk}}}}}}}$ with Gaussian distributions are also considered, and the results are qualitatively similar.

## Supplementary information

Supplementary Information

Description of Additional Supplementary Files

Supplementary Movie 1

Supplementary Movie 2

## Data Availability

The data that support the findings of this study are available from the corresponding author upon reasonable request.
