# Supplementary material for: Non-Hermitian physics for optical manipulation uncovers inherent instability of large clusters
Source: Nat Commun. 2021 Nov 15;12:6597. doi: 10.1038/s41467-021-26732-8 (PMC8593170; doi:10.1038/s41467-021-26732-8)
Supplement: Supplementary file 1 — Supplementary Information [file 41467_2021_26732_MOESM1_ESM.pdf]

Supplementary Information for

"Non-Hermitian Physics for Optical Manipulation Uncovers Inherent Instability of Large Clusters"

Xiao Li,[1,2] Yineng Liu,[3] Zhifang Lin,[4,5] Jack Ng,[1]* C. T. Chan,[2]

[1]*Department of Physics, Southern University of Science and Technology, Shenzhen, Guangdong 518055, China*

[2]*Department of Physics, The Hong Kong University of Science and Technology, Hong Kong, China*

[3]*Institute of Electromagnetics and Acoustics and School of Electronic Science and Engineering, Xiamen University, Xiamen 361005, China*

[4]*State Key Laboratory of Surface Physics, Key Laboratory of Micro and Nano Photonic Structures and Department of Physics, Fudan University, Shanghai, China*

[5]*Collaborative Innovation Center of Advanced Microstructures, Nanjing University, Nanjing, China*

*E-mail: wuzh3@sustech.edu.cn

Supplementary Note 1: Stability Analysis for Optical Trapping and Binding

Intense laser fields confine small particles at intensity extrema, a phenomenon known as optical trapping. Multiple particles can also be coupled together through light scattering, which modifies the spatial light distribution, thereby inducing a new particle-particle interaction known as optical binding.

Optical trapping and binding drive moving particles towards an equilibrium where they experience zero net force. Nonetheless, the existence of equilibrium does not necessarily imply stability. Upon perturbation, the particles will return to a stable equilibrium, but they will escape from an unstable equilibrium. Rigorous stability analysis is therefore essential, and is given below.

Particles illuminated by light and immersed in a fluid are subjected to optical forces, dissipative ambient damping, hydrodynamic couplings [1], and Brownian fluctuations. In this Supplementary Note 1, to obtain the analytical solutions, we focus on well-separated micro-particles illuminated by a laser field with modest intensity ($I_0 = 1.0$ mW/μm^2), such that the hydrodynamic coupling and Brownian motion can be neglected. For particles near each other, the hydrodynamic coupling will be considered in the numerical simulation presented in Supplementary Note 4.

Considering a cluster of *N* particles in a fluid, the equation of motion with hydrodynamic coupling and Brownian motion neglected is given by [2]

$$m\frac{d^2\Delta\mathbf{X}}{dt^2} = \mathbf{F}(\Delta\mathbf{X}) - \gamma\frac{d\Delta\mathbf{X}}{dt} \ , \tag{S1.1}$$

where m is the single-particle mass, $\mathbf{F} = \left(F_{x_1}, F_{y_1}, F_{z_1}, \cdots, F_{x_N}, F_{y_N}, F_{z_N}\right)$ are the optical forces, $\Delta\mathbf{X} = \left(\Delta x_1, \Delta y_1, \Delta z_1, \cdots, \Delta x_N, \Delta y_N, \Delta z_N\right)$ are the displacements from the equilibrium, $\gamma = 6\pi\eta r_S$ is the friction coefficient, η is the viscosity, r_S is the particle radius, and *t* is the time. According to the Lyapunov stability theorem [3], the stability of Eq. (S1.1) near an equilibrium is equivalent to its linear approximation:

$$m\frac{d^2\Delta\mathbf{X}}{dt^2} = \overleftrightarrow{\mathbf{K}} \cdot \Delta\mathbf{X} - \gamma\frac{d\Delta\mathbf{X}}{dt} \ , \tag{S1.2}$$

where $\overleftrightarrow{\mathbf{K}}_{ij} = \partial \mathbf{F}_i / \partial \Delta \mathbf{X}_j$ is the force matrix, and $\overleftrightarrow{\mathbf{K}} \cdot \Delta \mathbf{X}$ is the linear approximation to $\mathbf{F}$.[1]

Everywhere but at the exceptional point in which $\overleftrightarrow{\mathbf{K}}$ is defective, Eq. (S1.2) can be transformed by a similarity transformation using $\overleftrightarrow{\mathbf{V}}$ whose columns are the eigenvectors of $\overleftrightarrow{\mathbf{K}}$, and Eq. (S1.2) is reduced to a set of decoupled 2nd order differential equations:

$$m\frac{d^2\zeta_i}{dt^2} \approx K_i \cdot \zeta_i - \gamma \frac{d\zeta_i}{dt}, \tag{S1.3}$$

where K_i is the ith eigenvalue of $\overleftrightarrow{\mathbf{K}}$, i.e. $\left(\overleftrightarrow{\mathbf{V}}^{-1}\overleftrightarrow{\mathbf{K}}\overleftrightarrow{\mathbf{V}}\right)_{ij} = K_i\delta_{ij}$, and $\zeta_i = \sum_i \left(\overleftrightarrow{\mathbf{V}}^{-1}\right)_{ij} \Delta \mathbf{X}_j$. Equation (S1.3) can be solved using the standard substitution technique:

$$\zeta_i = \zeta_{0i} e^{i\Omega_i t}, \tag{S1.4}$$

where ζ_{0i} is a constant independent of time. The solution to Eq. (S1.3) can be categorized according to K_i, or equivalently according to the natural frequency of the eigenmode, given by

$$\Omega_i = \sqrt{-K_i / m}. \tag{S1.5}$$

In the following, we discuss several stability categories that depend upon the nature of K_i.

---

[1] For some optically-bound clusters consider in the main text, which are trapped on a plane of uniform intensity, those that do not possess spatial inversion symmetry are in a dynamical equilibrium such that the optical forces acting on all particles are the same but not zero. The cluster is therefore moving with a fixed shape. Eq. (S1.2) is generalized to $md^2\Delta\mathbf{X}/dt^2 = \mathbf{F_0} + \overleftrightarrow{\mathbf{K}} \cdot \Delta\mathbf{X} - \gamma d\Delta\mathbf{X}/dt$, where $\mathbf{F_0} = \left(F_{0x}, F_{0y}, F_{0z}, \cdots, F_{0x}, F_{0y}, F_{0z}\right)$ is a constant force acting on every particle. One may mathematically eliminate $\mathbf{F_0}$ and restore the equation of motion to the standard form of Eq. (S1.2) by applying the transformation: $\Delta\mathbf{x}' = \begin{cases} \Delta\mathbf{x} - \mathbf{F}_0 t/\gamma & b \neq 0 \\ \Delta\mathbf{x} - \mathbf{F}_0 t^2/2m & b = 0 \end{cases}$ [2].

a) Neutral mode characterized by K_i =0 (or Ω_i =0).

The forces acting on particles trapped on a plane of uniform intensity depend only on their relative positions. Consequently, there must be 2 neutral modes characterized by $K_\mathrm{i} = 0$, corresponding to a uniform translational motion along the trapping plane (assumed here to be the *xy*-plane):

$$\begin{aligned} \Delta X_\mathrm{x}(t) &= \left(1,0,0,\ldots,1,0,0,\ldots,1,0,0\right) \times (\alpha_\mathrm{x} + \beta_\mathrm{x} e^{-\gamma t/m}), \\ \Delta X_\mathrm{y}(t) &= \left(0,1,0,\ldots,0,1,0,\ldots,0,1,0\right) \times (\alpha_\mathrm{y} + \beta_\mathrm{y} e^{-\gamma t/m}), \end{aligned} \qquad \text{(S1.6)}$$

when α_x, β_x, α_y, and β_y are constants to be determined by the initial conditions.

b) Unstable mode characterized by a positive K_i (or an imaginary Ω_i).

$$\Delta \mathbf{X}_\mathrm{i}(t) = e^{-\gamma t/2m} \mathbf{v}_\mathrm{i} \times \left[\alpha_\mathrm{i} e^{-t\sqrt{(\gamma/2m)^2 + |\Omega_\mathrm{i}|^2}} + \beta_\mathrm{i} e^{t\sqrt{(\gamma/2m)^2 + |\Omega_\mathrm{i}|^2}} \right],$$

For $K_\mathrm{i} > 0$, Ω_i is purely imaginary and the eigenmode is

(S1.7)

where $\mathbf{v}_\mathrm{i}$ is the ith eigenvector of K_i, and α_i and β_i are constants to be determined by the initial conditions. Eq. (S1.7) diverges with time, and the mode is thus unstable.

c) Stable mode characterized by a negative K_i (or a real Ω_i).

For $K_\mathrm{i} < 0$, one has harmonic oscillation-like modes:

$$\Delta \mathbf{X}_\mathrm{i}(t) = \begin{cases} e^{-\gamma t/2m} \mathbf{v}_\mathrm{i} \times \left[\alpha_\mathrm{i} e^{-t\sqrt{(\gamma/2m)^2 - \Omega_\mathrm{i}^2}} + \beta_\mathrm{i} e^{t\sqrt{(\gamma/2m)^2 - \Omega_\mathrm{i}^2}} \right] & \text{if } (\gamma/2m)^2 > \Omega_\mathrm{i}^2 \text{ (over damped)} \\ e^{-\gamma t/2m} \mathbf{v}_\mathrm{i} \times \left[\alpha_\mathrm{i} + \beta_\mathrm{i} t \right] & \text{if } (\gamma/2m)^2 = \Omega_\mathrm{i}^2 \text{ (critically damped)}, \\ A_\mathrm{i} e^{-\gamma t/2m} \mathbf{v}_\mathrm{i} \times \sin\left[\sqrt{\Omega_\mathrm{i}^2 - (\gamma/2m)^2}\, t + \varphi_\mathrm{i} \right] & \text{if } (\gamma/2m)^2 < \Omega_\mathrm{i}^2 \text{ (under damped)} \end{cases} \qquad \text{(S1.8)}$$

where α_i and β_i or A_i and φ_i are constants to be determined by the initial conditions. All these modes are stable.

d) Complex mode characterized by a complex K_i (or a complex Ω_i).

Optical forces are open and thus nonconservative. Consequently, $\overleftrightarrow{\mathbf{K}} \neq \overleftrightarrow{\mathbf{K}}^{\mathrm{T}}$ such that complex conjugate pairs of eigenvalues are allowed, even for a real matrix $\overleftrightarrow{\mathbf{K}}$: $K_{\mathrm{i}+1} = K_{\mathrm{i}}^*$. The modes corresponding to the conjugate pair of eigenvalues are

$$\begin{aligned} \Delta\mathbf{X}_{\mathrm{i}+}(t) &= \alpha_{\mathrm{i}} e^{-\mathrm{Im}(\Omega_{\mathrm{i}+})t} \times \left\{ \mathrm{Re}[\mathbf{v}_{\mathrm{i}}]\cos\left(\mathrm{Re}[\Omega_{\mathrm{i}+}]t + \varphi_{\mathrm{i}\alpha}\right) - \mathrm{Im}[\mathbf{v}_{\mathrm{i}}]\sin\left(\mathrm{Re}[\Omega_{\mathrm{i}+}]t + \varphi_{\mathrm{i}\alpha}\right)\right\}, \\ \Delta\mathbf{X}_{\mathrm{i}-}(t) &= \beta_{\mathrm{i}} e^{-\mathrm{Im}(\Omega_{\mathrm{i}-})t} \times \left\{ \mathrm{Re}[\mathbf{v}_{\mathrm{i}}]\cos\left(\mathrm{Re}[\Omega_{\mathrm{i}-}]t + \varphi_{\mathrm{i}\beta}\right) + \mathrm{Im}[\mathbf{v}_{\mathrm{i}}]\sin\left(\mathrm{Re}[\Omega_{\mathrm{i}-}]t + \varphi_{\mathrm{i}\beta}\right)\right\}, \end{aligned} \tag{S1.9}$$

where α_{i}, β_{i}, $\varphi_{\mathrm{i}\alpha}$, and $\varphi_{\mathrm{i}\beta}$ are constants to be determined by the initial conditions,

$$\begin{aligned} \mathrm{Re}(\Omega_{\mathrm{i}\pm}) &= \mp\left[(\gamma^2 + 4m\,\mathrm{Re}[K_{\mathrm{i}}])^2 + 16m^2\,\mathrm{Im}[K_{\mathrm{i}}]^2\right]^{1/4} \sin\left(\delta_{\mathrm{i}}/2\right)/2m, \\ \mathrm{Im}(\Omega_{\mathrm{i}\pm}) &= \gamma \pm \left[(\gamma^2 + 4m\,\mathrm{Re}[K_{\mathrm{i}}])^2 + 16m^2\,\mathrm{Im}[K_{\mathrm{i}}]^2\right]^{1/4} \cos\left(\delta_{\mathrm{i}}/2\right)/2m, \end{aligned} \tag{S1.10}$$

and

$$\delta_{\mathrm{i}} = \begin{cases} \tan^{-1}\dfrac{4m\left|\mathrm{Im}[K_{\mathrm{i}}]\right|}{\gamma^2 + 4m\,\mathrm{Re}[K_{\mathrm{i}}]} & \text{if} \quad \gamma^2 + 4m\,\mathrm{Re}[K_{\mathrm{i}}] > 0, \\ \pi - \tan^{-1}\dfrac{4m\left|\mathrm{Im}[K_{\mathrm{i}}]\right|}{\left|\gamma^2 + 4m\,\mathrm{Re}[K_{\mathrm{i}}]\right|} & \text{if} \quad \gamma^2 + 4m\,\mathrm{Re}[K_{\mathrm{i}}] < 0. \end{cases} \tag{S1.11}$$

The stabilities of the complex modes are summarized in Supplementary Fig. 1. For $\mathrm{Re}[K_{\mathrm{i}}] > 0$, conservative and nonconservative forces both induce instability. The former corresponds to a potential energy maximum, while the latter will induce a centrifugal force that tears the partiles away. Thus the cluster is unstable. For $\mathrm{Re}[K_{\mathrm{i}}] < 0$ and $\gamma < \gamma_{\mathrm{critical}} = \sqrt{m}\left|\mathrm{Im}[K_{\mathrm{i}}]\right| / \sqrt{\left|\mathrm{Re}[K_{\mathrm{i}}]\right|}$, the conservative force attracts particles toward the equilibrium, however, the nonconservative force causes the particles to revolve with increasing speed, eventually overcoming any attraction. In contrast, if $\mathrm{Re}[K_{\mathrm{i}}] < 0$ and $\gamma > \gamma_{\mathrm{critical}}$, the damping is sufficient to remove the kinetic energy pumped in by the nonconservative force, thereby maintaining the stability of the cluster. See main text for further details.

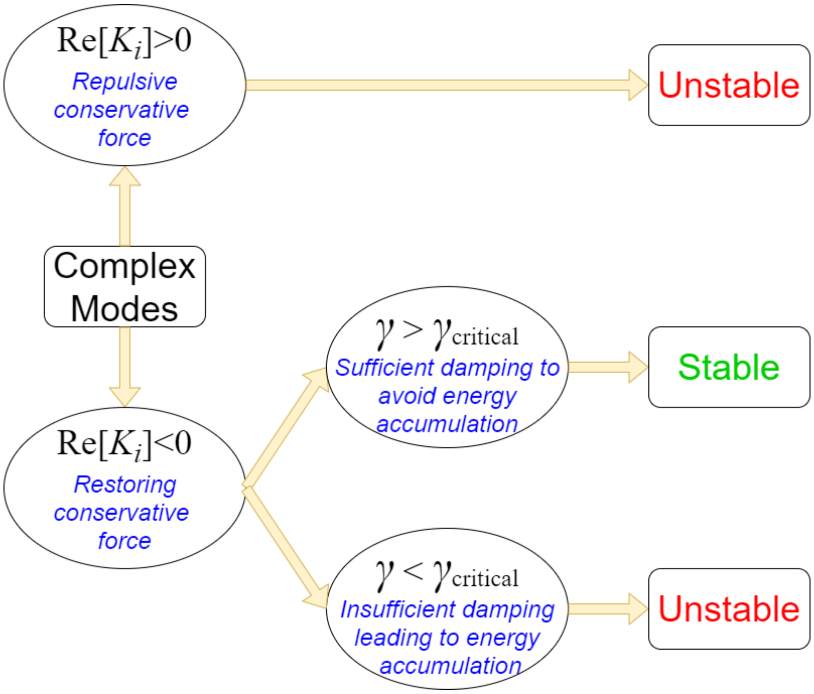

Supplementary Fig. 1| **Flow-chart illustrating the stability of complex modes.**

e) Stability at Exceptional Points (EPs)

For completeness, we present the stability analysis for a system at its exceptional point (EP), where particles are affected solely by optical forces.

For simplicity, we consider an arbitrary 2×2 force matrix. The treatment for matrices with arbitrary sizes is available in Ref. [3]. The force matrix at the EP, after appropriate rotation, can be written as

$$\overleftrightarrow{\mathbf{K}} = \begin{bmatrix} a+b & g \\ -g & a-b \end{bmatrix} = \begin{bmatrix} a+g & g \\ -g & a-g \end{bmatrix}, \tag{S1.12}$$

where $b = g$ at the EP and $\overleftrightarrow{\mathbf{K}}$ becomes defective. Both eigenvalues are a, but there is only one eigenvector

$$\mathbf{v}_1 = \begin{bmatrix} -1 \\ 1 \end{bmatrix}. \tag{S1.13}$$

The generalized eigenvector $\mathbf{u}_1$, corresponding to $\mathbf{v}_1$, can be obtained by solving

$$\left(\overleftrightarrow{\mathbf{K}} - a\overleftrightarrow{\mathbf{I}}\right)\mathbf{u}_1 = \mathbf{v}_1, \tag{S1.14}$$

which gives

$$\mathbf{u}_1 = \begin{bmatrix} -1/g \\ 0 \end{bmatrix}. \tag{S1.15}$$

The eigenmodes at the EP are given by

$$\begin{cases} \Delta\mathbf{X}_1 = \alpha_1 \mathbf{v}_1 e^{i\sqrt{\frac{-a}{m}}t}, \\ \Delta\mathbf{X}_2 = \alpha_2 (t\mathbf{v}_1 + \mathbf{u}_1) e^{i\sqrt{\frac{-a}{m}}t}, \end{cases} \tag{S1.16}$$

where α_1 and α_2 are constants to be determined by the initial conditions. In all cases, the second mode diverges with time, and therefore the cluster is unstable.

Supplementary Note 2: Symmetry and Nonconservative Forces

In general, $\overleftrightarrow{\mathbf{K}}$ is asymmetric for nonconservative systems, such as optical trapping and binding. However, sometimes some asymmetric components in $\overleftrightarrow{\mathbf{K}}$ are incompatible with the system symmetry, and must vanish. This is especially important for small clusters. Consider the pair of spheres shown in Fig. 2(b) of the main text. To be specific, we assume $n_{\mathrm{r}} = 1.2$, $\lambda = 1064$ nm and $r_{\mathrm{S}} = 0.41\lambda$, but the conclusion drawn is independent of these details. The force matrix turns out to be symmetric:

$$\overleftrightarrow{\mathbf{K}} = \begin{bmatrix} 0.02682 & 0 & -0.02682 & 0 \\ 0 & -0.57957 & 0 & 0.57957 \\ -0.02682 & 0 & 0.02682 & 0 \\ 0 & 0.57957 & 0 & -0.57957 \end{bmatrix} \frac{\mathrm{pN}}{\mu\mathrm{m}}, \tag{S2.1}$$

due to mirror symmetries about the *xz* and *yz* planes. To see this, consider Supplementary Fig. 2(a). On one hand, the small displacements Δx (red) and $-\Delta x$ (black) induce *y*-directional forces of $\left(\partial F_{\mathrm{y}_1} / \partial x_1\right)\Delta x$ and $-\left(\partial F_{\mathrm{y}_1} / \partial x_1\right)\Delta x$ on the first (top) particle, respectively. On the other hand, the *yz*-plane mirror symmetry requires the induced forces to be equal. Accordingly, the only solution is $\partial F_{\mathrm{y}_1} / \partial x_1 = 0$, represented by the red zero in Eq. (S2.1). Similarly, in Supplementary Fig. 2(b), the red and blue forces are induced by the red and blue Δx, respectively, and are thus equal due to the mirror symmetry, which are represented by the two blue components in Eq. (S2.1). Other symmetries may be considered in a similar manner, which eventually resulted in the symmetric matrix Eq. (S2.1).

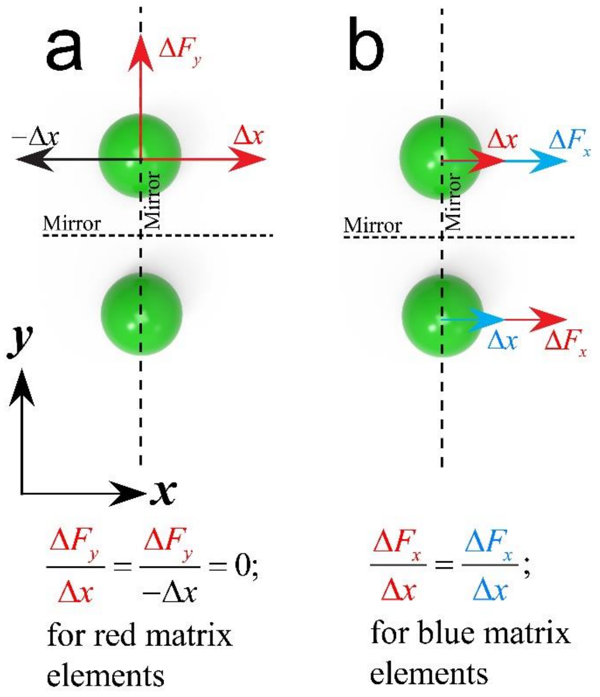

Supplementary Fig. 2| **Schematics illustrating how the symmetries of the cluster affect the matrix elements in Eq. (S2.1).**

The 3-sphere triangle shown in Supplementary Fig. 3 is different from the 2-sphere cluster in that it has fewer symmetries and more degrees of freedom. Each particle adds 2 degrees of freedom on the *xy*-plane, which gives a 6×6 force matrix, and thus 6 modes in total. The force matrix, when $n_{\mathrm{r}} = 1.2$ and $r_{\mathrm{S}} = 0.41\lambda$, is

$$\overleftrightarrow{\mathbf{K}} = \begin{bmatrix} -0.02209 & 0 & 0.01104 & 0.00896 & 0.01104 & -0.00896 \\ 0 & -0.17777 & -0.07155 & 0.08888 & 0.07155 & 0.08888 \\ 0.01225 & 0.00780 & -0.43439 & -0.00703 & 0.42214 & -0.00077 \\ -0.08343 & 0.09331 & 0.07258 & -0.08694 & 0.01087 & -0.00636 \\ 0.01225 & -0.00780 & 0.42214 & 0.00077 & -0.43439 & 0.00703 \\ 0.08343 & 0.09331 & -0.01087 & -0.00636 & -0.07258 & -0.08694 \end{bmatrix} \frac{\mathrm{pN}}{\mu\mathrm{m}}. \quad \text{(S2.2)}$$

The system is conservative if and only if $\overleftrightarrow{\mathbf{A}}$, the anti-symmetric part of $\overleftrightarrow{\mathbf{K}}$, vanishes

$$\overleftrightarrow{\mathbf{A}} = \frac{\overleftrightarrow{\mathbf{K}} - \overleftrightarrow{\mathbf{K}}^{\mathrm{T}}}{2}$$

$$= \begin{bmatrix} 0 & 0 & -0.00061 & 0.04620 & -0.00061 & -0.04620 \\ 0 & 0 & -0.03968 & -0.00222 & 0.03968 & -0.00222 \\ 0.00061 & 0.03968 & 0 & -0.03981 & 0 & 0.00505 \\ -0.04620 & 0.00222 & 0.03981 & 0 & 0.00505 & 0 \\ 0.00061 & -0.03968 & 0 & -0.00505 & 0 & 0.03981 \\ 0.04620 & 0.00222 & -0.0051 & 0 & -0.03981 & 0 \end{bmatrix} \frac{\mathrm{pN}}{\mu\mathrm{m}}. \quad \text{(S2.3)}$$

It is noteworthy that the *yz* mirror symmetric plane has forced some components of $\overleftrightarrow{\mathbf{A}}$ to vanish. Yet, the limited symmetries are still insufficient to make $\overleftrightarrow{\mathbf{K}}$ symmetric, and thus the system is nonconservative. Similarly, for the configuation in Fig. 2(c) with two mirror symmetries, when $n_{\mathrm{r}} = 1.2$ and $r_{\mathrm{S}} = 0.41\lambda$, the force matrix is

$$\overleftrightarrow{\mathbf{K}} = \begin{bmatrix} 0.00980 & 0 & -0.00604 & 0 & -0.00376 & 0 \\ 0 & -0.15930 & 0 & 0.14001 & 0 & 0.01928 \\ -0.00734 & 0 & 0.01468 & 0 & -0.00734 & 0 \\ 0 & 0.13882 & 0 & -0.27764 & 0 & 0.13882 \\ -0.00376 & 0 & -0.00604 & 0 & 0.00980 & 0 \\ 0 & 0.01928 & 0 & 0.14001 & 0 & -0.15930 \end{bmatrix} \frac{\mathrm{pN}}{\mu\mathrm{m}}. \quad \text{(S2.4)}$$

The corresponding asymmetric part is

$$\overleftrightarrow{\mathbf{A}} = \begin{bmatrix} 0 & 0 & 0.00065 & 0 & 0 & 0 \\ 0 & 0 & 0 & 0.00060 & 0 & 0 \\ -0.00065 & 0 & 0 & 0 & -0.00065 & 0 \\ 0 & -0.00060 & 0 & 0 & 0 & -0.00060 \\ 0 & 0 & 0.00065 & 0 & 0 & 0 \\ 0 & 0 & 0 & 0.00060 & 0 & 0 \end{bmatrix} \frac{\mathrm{pN}}{\mu\mathrm{m}}. \quad \text{(S2.5)}$$

Because two mirror symmetries are present, there are more zeros in $\overleftrightarrow{\mathbf{K}}$ and $\overleftrightarrow{\mathbf{A}}$. However, the system is still nonconservative. In Supplementary Fig. 4, we explained the inequality of the blue elements in Eq. (S2.4). The lack of mirror symmetries between the top and middle spheres is the reason for the asymmetry of $\overleftrightarrow{\mathbf{K}}$ in Eq. (S2.4).

3 Spheres:

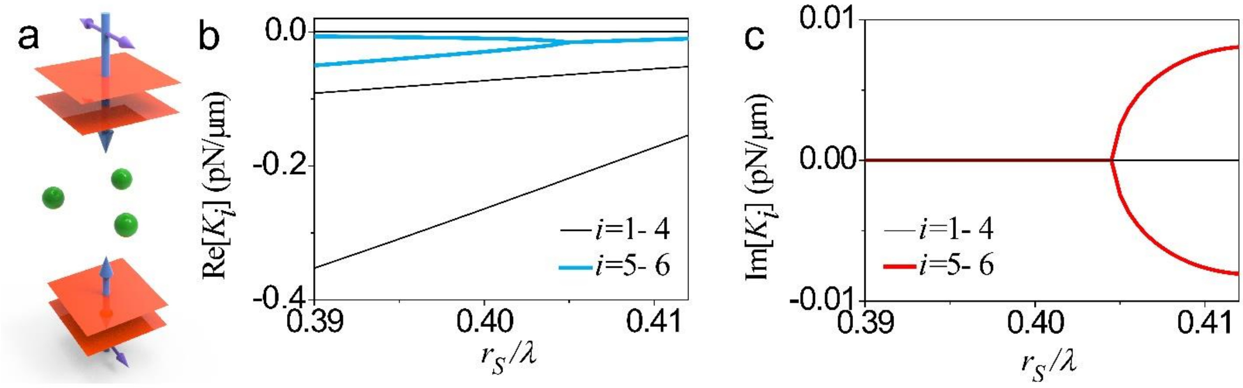

Supplementary Fig. 3| **Three-sphere triangular cluster bound by light.** (a) A schematic plot for optical binding of 3 spheres, with relative refractive index $n_r = 1.2$. (b-c) The real (b) and imaginary (c) parts of K_i versus particle radius. The modes that make a transition to complex modes are highlighted with light blue and red.

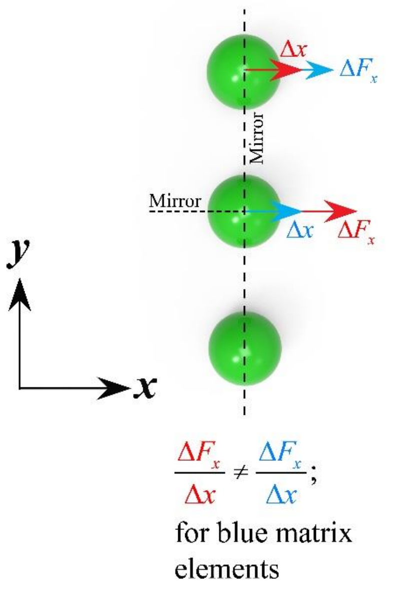

Supplementary Fig. 4| **Schematics illustrating how the symmetries of the cluster affect the matrix elements in Eq. (S2.4).** The inequality shown in the Figure is a result of lacking mirror symmetry between the top and middle spheres.

Four different 4-sphere configurations are shown in Supplementary Fig. 5. The physics of these is similar to that of the 3-sphere clusters.

Supplementary Fig. 6 shows the eigenvalues for the configuration shown in the inset of Supplementary Fig. 6(c), where 25 particles are trapped by a square optical lattice formed by a standing wave, and the particles are bound by scattering. Supplementary Fig. 6(a)-(b) and Fig. 6(c)-(d) are, respectively, the eigenvalues on linear and logarithmic scales. For Rayleigh particles illuminated by a standing wave, optical trapping and optical binding are approximately conservative [4]. The eigenvalues are real for a small radius. However, the nonconservative force increases at a faster rate and induces complex eigenvalues beginning at a radius of approximately one-sixth of a wavelength. More complex eigenvalues emerge later, and a total of 10 complex modes occur out of 50 transverse modes in the parameter range considered. We note that the steep increase of the imaginary components near the EP may seem to contradict the square-root-increase law for a second-order EP, but if one zooms into the exceptional point, one will see that the square-root-increase law still holds.

4 Spheres:

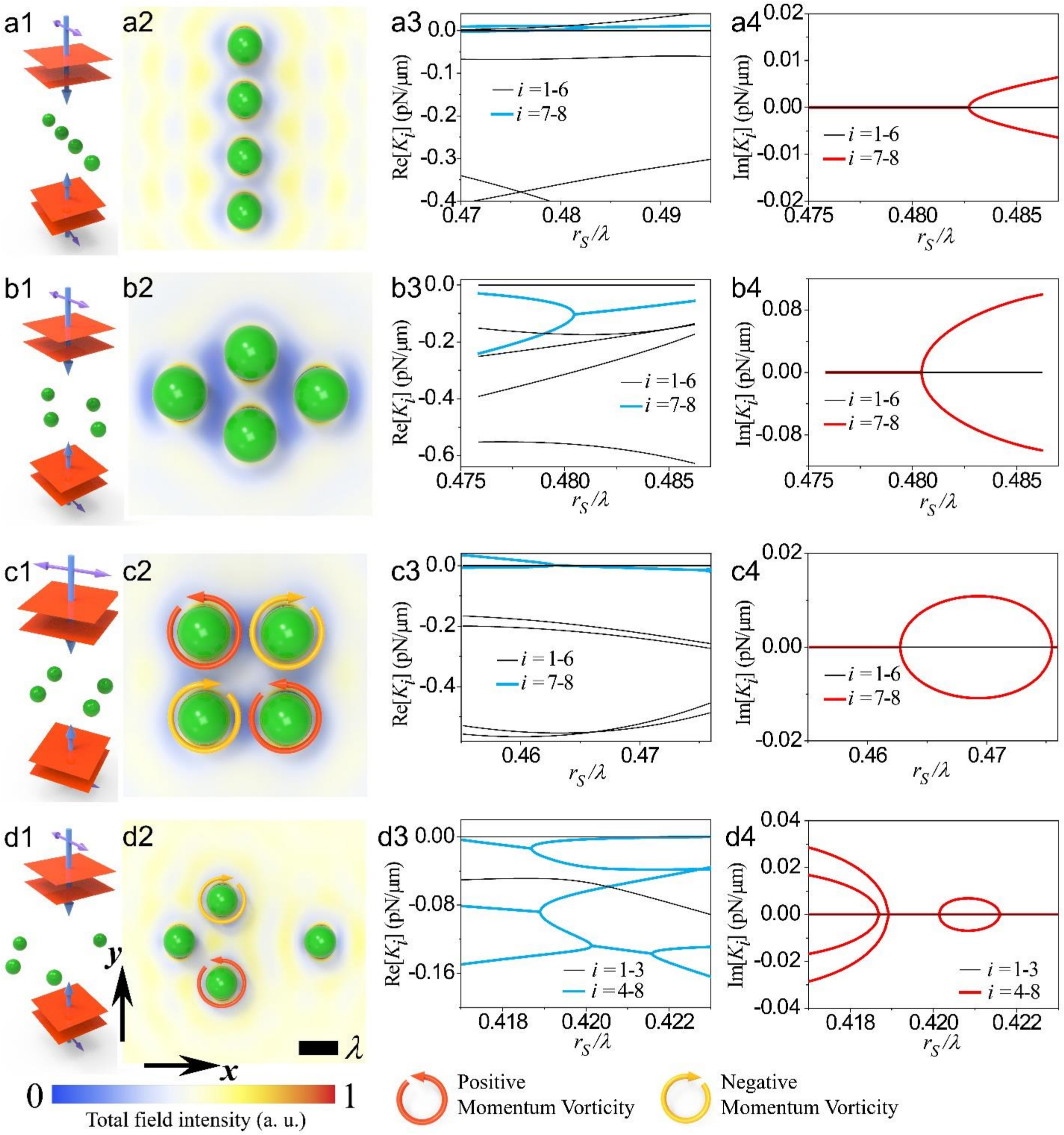

Supplementary Fig. 5| **Four-sphere clusters bound by light.** (a1-a4) A chain with $n_r = 1.30$. (b1-b4) A rhombus with $n_r = 1.30$. (c1-c4) A rectangle with $n_r = 1.25$. (d1-d4) A kite with $n_r = 1.25$. The red and yellow arrows denote the direction of momentum vorticity, defined in the main text.

25 Spheres:

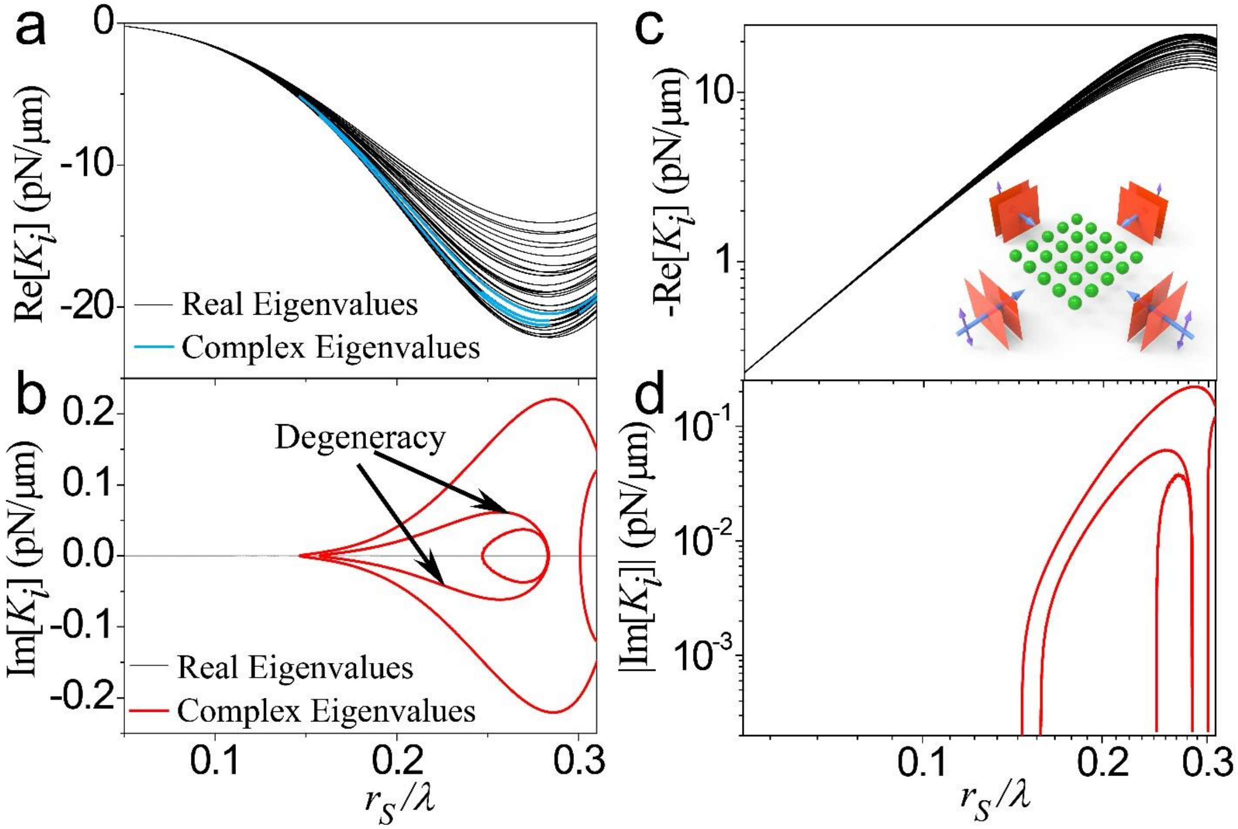

Supplementary Fig. 6| K_{i} **for the 25-sphere optically-bound square lattice illustrated in the inset of (c).** Two pairs of counter-propagating plane waves, forming a standing wave in the shape of a square lattice, trap and bind the 25 particles ($n_{\mathrm{r}} = 1.2$). The real (a) and imaginary (b) parts of the 50 in-plane eigenvalues of the force matrix are plotted in (a) and (b), respectively. The real and imaginary parts of the 10 complex eigenvalues are highlighted in light blue and red, respectively. The degenerate modes are marked in (b). The eigenvalues are plotted on a logarithmic scale in (c-d).

Supplementary Note 3: Arrangement of Incident Waves and Particles

This supplementary note shows in detail the actual configuration considered in the main text. The optically-bound clusters are grouped into 3 classes: ***Lattices Bound by Standing Wave***, ***Lattices Bound by Non-Standing Wave***, and ***Planar Optically Bound Clusters*** (see the directory below for details).

Lattices Bound by Standing Wave and ***Lattices Bound by Non-Standing Wave*** refer to lattices of particles trapped by a periodic (or quasi-periodic) interference pattern formed by a few coherent plane waves, with the former being a standing incident wave, while the latter being a non-standing incident wave. For small to moderately sized clusters, the trapping force is much larger than the binding force. The particles are trapped by the hotspots of the interference patterns, while the optical binding force slightly perturbs the positions of the trapped particles. In Supplementary Fig. 7 both the average distance of the particles from the lattice hotspots $\langle d_{\mathrm{i}} \rangle = N^{-1}\sum_{\mathrm{i}=1}^{N} d_{\mathrm{i}}$ and its standard deviation $\sqrt{\langle d_{\mathrm{i}}^2 \rangle} = \sqrt{N^{-1}\sum_{\mathrm{i}=1}^{N} d_{\mathrm{i}}^2}$ are plotted for a 7×7 array. The lattice constant is approximately λ. The deviations of the particles from the hotspots are quite small even for near-contact particles, indicating the dominance of the trapping force. Surprisingly, the much weaker optical binding force plays a significant role in stability in the many-particle limit, as discussed in the main text.

For ***Planar Optically Bound Clusters***, the particles are confined on the *xy*-plane by two counter-propagating plane waves and then bound together by the scattered light. In such a structure the transverse binding arises solely from the optical binding force. We start with an arbitrarily chosen configuration, and then apply our molecular simulation technique to search for the equilibrium positions.

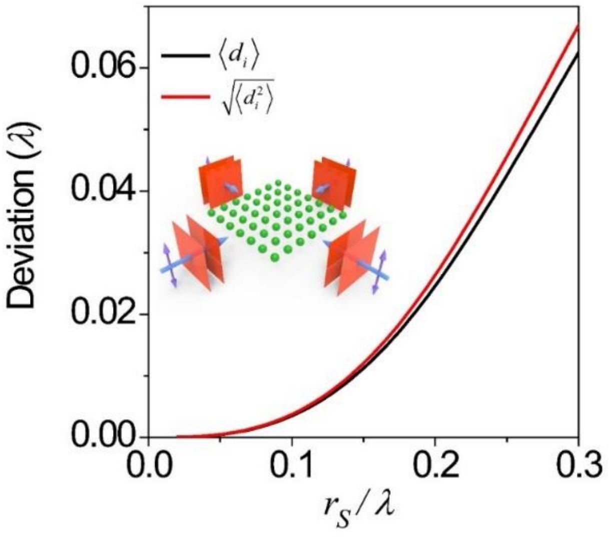

Supplementary Fig. 7| **The averaged distance of the particle equilibriums from the hotspots and its standard derivation, for the** 7×7 **square lattice shown in the inset.** Parameters and other details of the optically trapped and bound structures are consistent with Fig. 3 in the main text.

Directory

Lattices Bound by Standing Wave

Planar square lattices ....Fig. 3(b)

Cubic lattices (3D) .... Fig. 3(c)

Lattices Bound by Non-Standing Wave

Planar quasi-crystal lattices .... Fig. 3(e)

Planar triangular lattices A .... Fig. 3(f)

Planar triangular lattices B .... Fig. 3(g)

Planar Optically Bound Clusters

Planar optically bound clusters .... Fig. 3(d)

Lattices Bound by Standing Wave

Planar square lattices:

The optically bound square lattices with different numbers of particles are schematically illustrated in Supplementary Fig. 8. The incident field consists of two orthogonal pairs of counter-propagating plane waves:

$$\mathbf{E}_{\text{in}}(\mathbf{r}) = E_0\left(e^{ikx}\hat{\mathbf{z}} + e^{-ikx}\hat{\mathbf{z}} + e^{iky}\hat{\mathbf{z}} + e^{-iky}\hat{\mathbf{z}}\right) = 2E_0\hat{\mathbf{z}}\left(\cos kx + \cos ky\right), \tag{S3.1}$$

where $k = 2\pi/\lambda$ is the wavenumber. The lattice constant is λ.

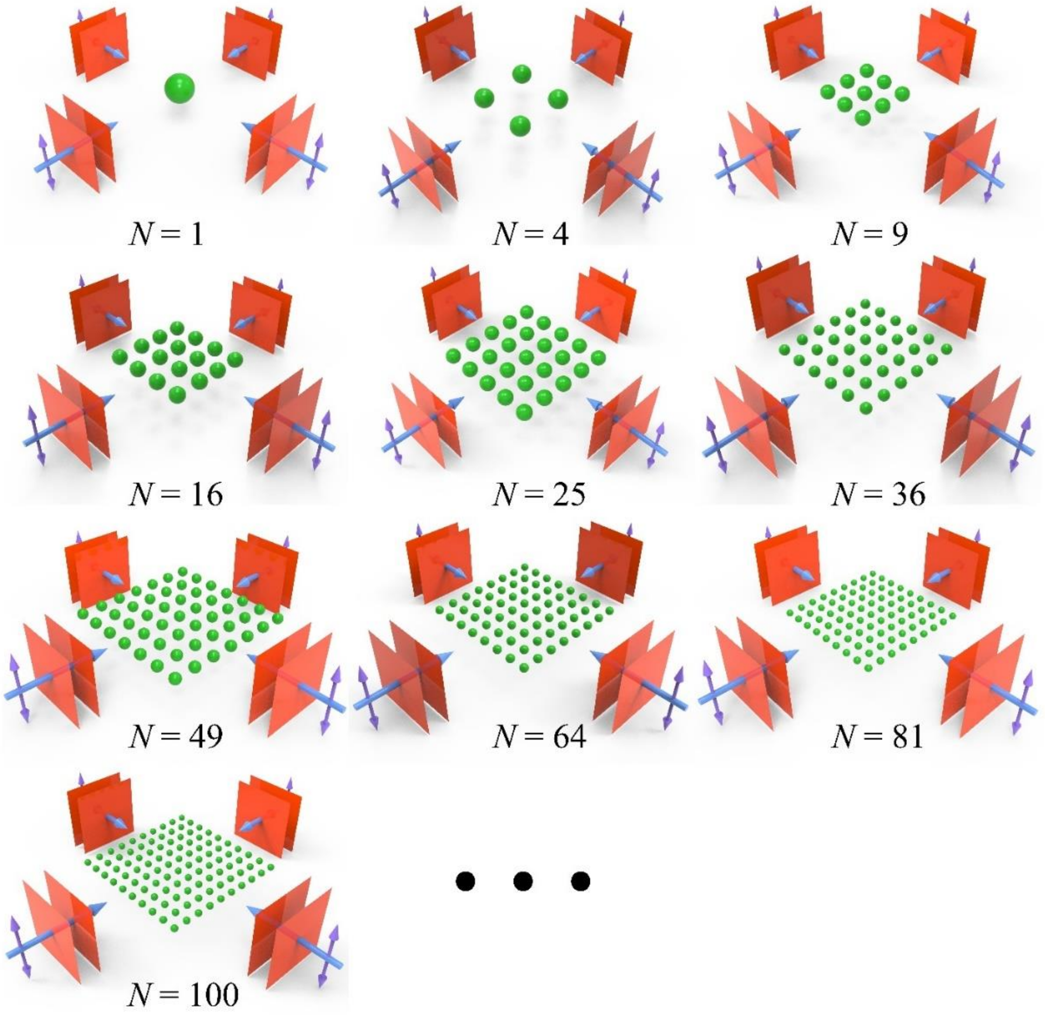

Supplementary Fig. 8| **Planar square lattices bound by standing wave.** The number of particles ranges from N = 1 to 289. Clusters with N > 100 are not shown.

Cubic lattices (3D):

The incident wave consists of three orthogonal pairs of counter-propagating plane waves (Supplementary Fig. 9):

$$\begin{aligned}\mathbf{E}_{\text{in}}(\mathbf{r}) &= E_0\left(e^{ikx}\hat{\mathbf{z}} + e^{-ikx}\hat{\mathbf{z}} + e^{iky}\hat{\mathbf{z}} + e^{-iky}\hat{\mathbf{z}} + e^{ikz}\hat{\mathbf{x}} + e^{-ikz}\hat{\mathbf{x}}\right) \\ &= 2E_0\left(\hat{\mathbf{z}}\cos kx + \hat{\mathbf{z}}\cos ky + \hat{\mathbf{x}}\cos kz\right).\end{aligned} \quad \text{(S3.2)}$$

The lattice constant is approximately λ .

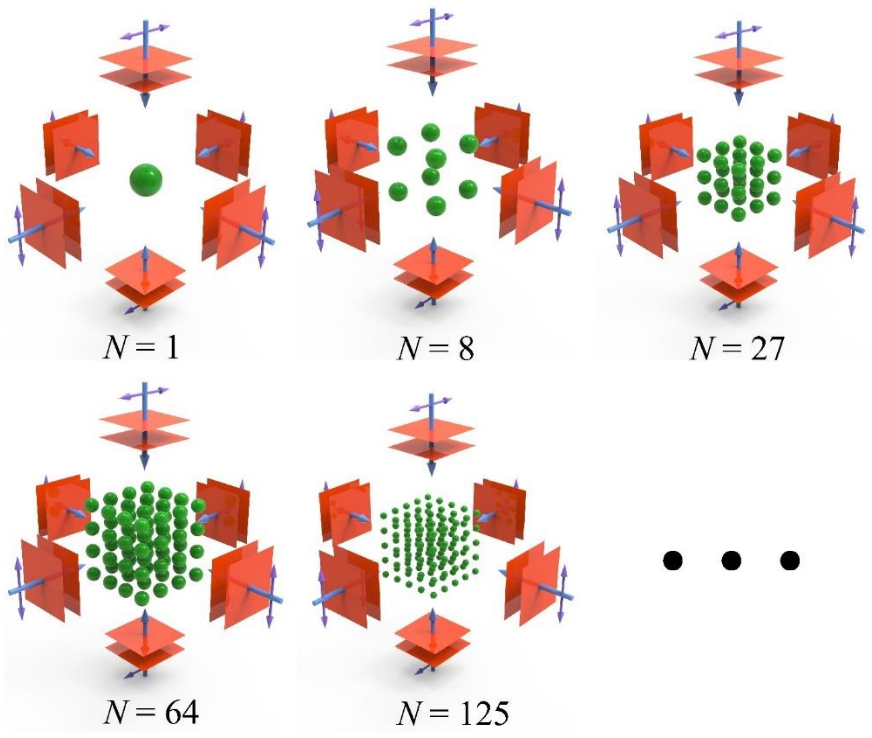

Supplementary Fig. 9| **Optically bound 3D cubic lattices.** The number of particles considered ranges from N = 1 to 729. Clusters with N > 125 are not shown.

Lattices Bound by Non-Standing Wave

Planar quasi-crystal lattices:

The incident wave consists of five equiangular plane waves, as depicted in Supplementary Fig. 10:

$$\mathbf{E}_{\text{in}}(\mathbf{r}) = E_0 \begin{pmatrix} e^{ikx}\hat{\mathbf{z}} + e^{ik\left(\cos\frac{2\pi}{5}x+\sin\frac{2\pi}{5}y\right)}\hat{\mathbf{z}} + e^{ik\left(\cos\frac{4\pi}{5}x+\sin\frac{4\pi}{5}y\right)}\hat{\mathbf{z}} \\ +e^{ik\left(\cos\frac{6\pi}{5}x+\sin\frac{6\pi}{5}y\right)}\hat{\mathbf{z}} + e^{ik\left(\cos\frac{8\pi}{5}x+\sin\frac{8\pi}{5}y\right)}\hat{\mathbf{z}} \end{pmatrix}. \tag{S3.3}$$

The hotspots resulted from the wave interference form a quasi-crystalline optical lattice. The particles are trapped near the hotspots. Supplementary Fig. 10 illustrates the clusters with different numbers of particles (N). The particles are introcuced in a ring-by-ring manner.

Layer No.	1	2	3	4	5	6	7	8	9
No. of Particles N	1	11	21	31	41	51	71	91	101

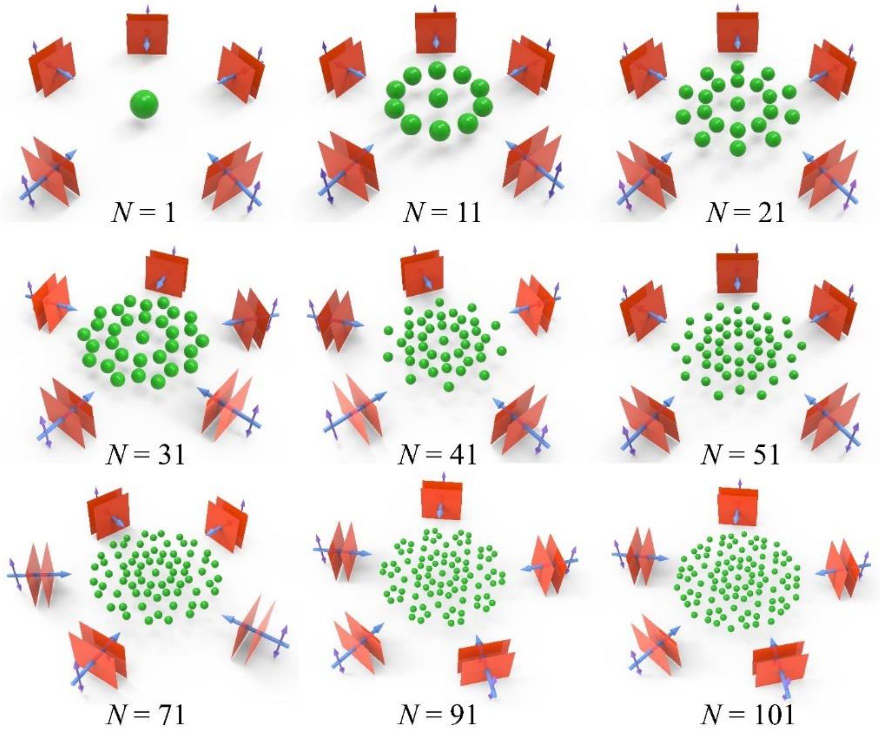

Supplementary Fig. 10| **Optically bound planar quasi-crystal lattices.** The number of particles considered ranges from N = 1 to 101.

Planar triangular lattices A:

The incident wave, as depicted in Supplementary Fig. 11, is given by:

$$\mathbf{E}_{\text{in}}(\mathbf{r}) = E_0 \left(e^{ikx}\hat{\mathbf{z}} + e^{ik\left(\cos\frac{2\pi}{3}x + \sin\frac{2\pi}{3}y\right)}\hat{\mathbf{z}} + e^{ik\left(\cos\frac{4\pi}{3}x + \sin\frac{4\pi}{3}y\right)}\hat{\mathbf{z}} \right). \tag{S3.4}$$

The lattice constant is approximately 0.7λ. The particles are introduced in a ring-by-ring manner, as illustrated in Supplementary Fig. 11.

Layer No. *m*	1	2	3	4	5	6	7
No. of Particles *N*	1	7	19	37	61	91	127

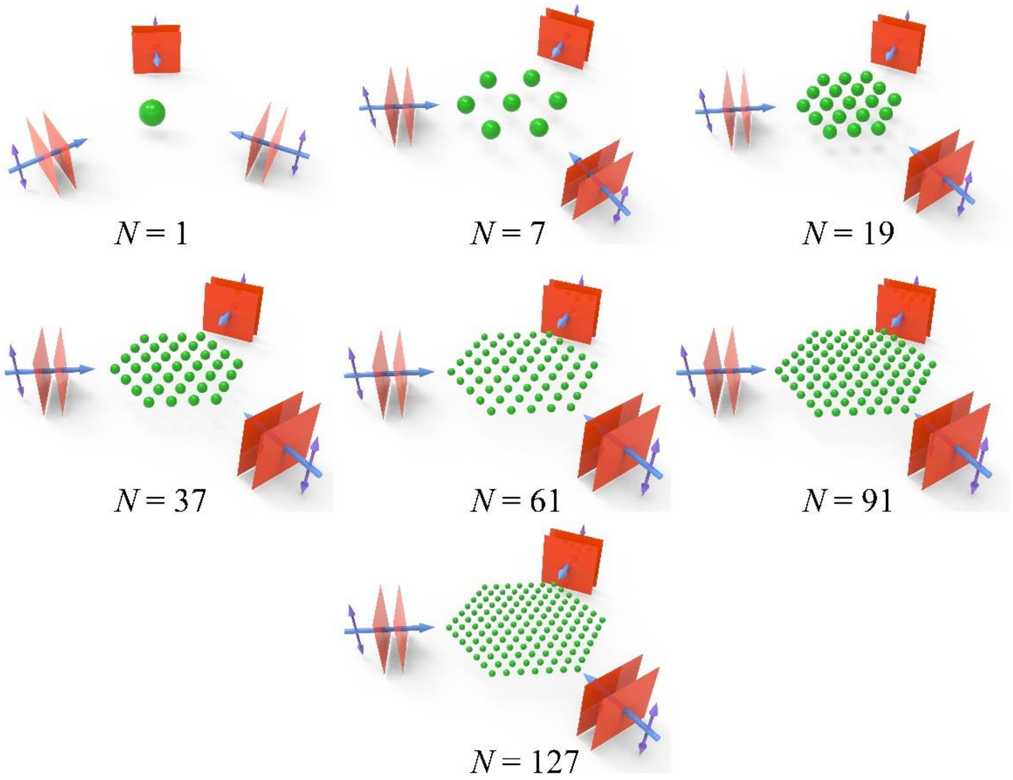

Supplementary Fig. 11| **Optically bound planar triangular lattices A.** The number of particles considered ranges from N = 1 to 127. The particles are introduced in a ring-by-ring manner.

Planar triangular lattices B:

In Supplementary Fig. 12, the incident wave is

$$\mathbf{E}_{\text{in}}(\mathbf{r}) = E_0 \begin{pmatrix} e^{ik\left(\sin(1^\circ)x+\cos(1^\circ)z\right)}\left(\cos(1^\circ)\hat{\mathbf{x}}-\sin(1^\circ)\hat{\mathbf{z}}\right) \\ +e^{ik\left(\cos\frac{2\pi}{3}\sin(1^\circ)x+\sin\frac{2\pi}{3}\sin(1^\circ)y+\cos(1^\circ)z\right)}\left(0.999962\hat{\mathbf{x}}+0.0087272\hat{\mathbf{z}}\right) \\ +e^{ik\left(\cos\frac{4\pi}{3}\sin(1^\circ)x+\sin\frac{4\pi}{3}\sin(1^\circ)y+\cos(1^\circ)z\right)}\left(0.999962\hat{\mathbf{x}}+0.0087272\hat{\mathbf{z}}\right) \end{pmatrix}, \qquad \text{(S3.5)}$$

The three incident plane waves are approximately *x*-polarized and each makes an angle of 1° with the *z*-axis (beams' propagating direction). In the simulation, we ignore the *z*-directional forces, while in the experiment in ref. [5], the particles are pushed onto a glass surface. The lattice constant is approximately $15\ \mu\text{m}$. The particles are introduced in a ring-by-ring manner, as shown in Supplementary Fig. 12.

Layer No. *m*	1	2	3	4	5	6
No. of Particles *N*	1	7	19	37	61	91

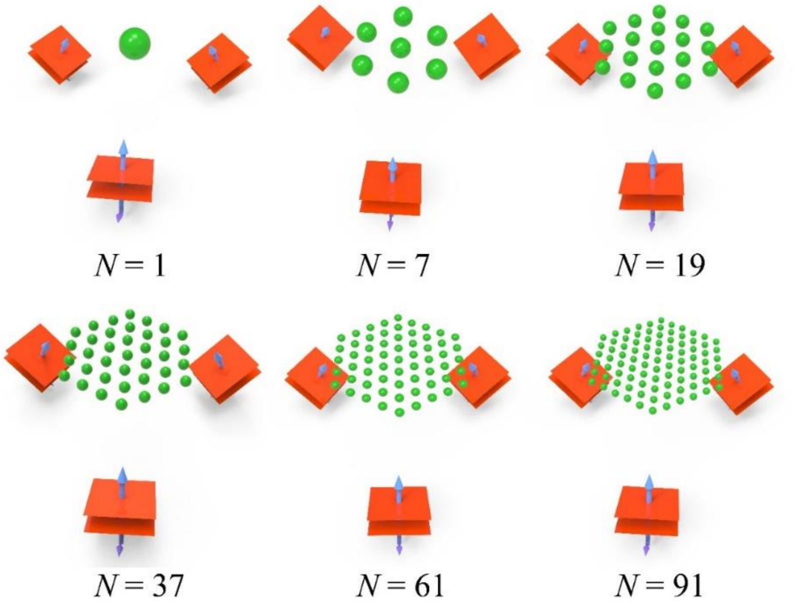

Supplementary Fig. 12| **Planar triangular lattice B (simulating experiments in Ref. [5]).** The number of particles considered ranges from $N = 1$ to 91. The particles (N) are introduced in a ring-by-ring manner.

Planar Optically Bound Clusters

The incident wave consists of two counter-propagating plane waves (y-polarized):

$$\mathbf{E}_{\text{in}}(\mathbf{r}) = E_0\left(e^{ikz}\hat{\mathbf{y}} + e^{-ikz}\hat{\mathbf{y}}\right) = 2E_0\hat{\mathbf{y}}\cos kz\ . \tag{S3.6}$$

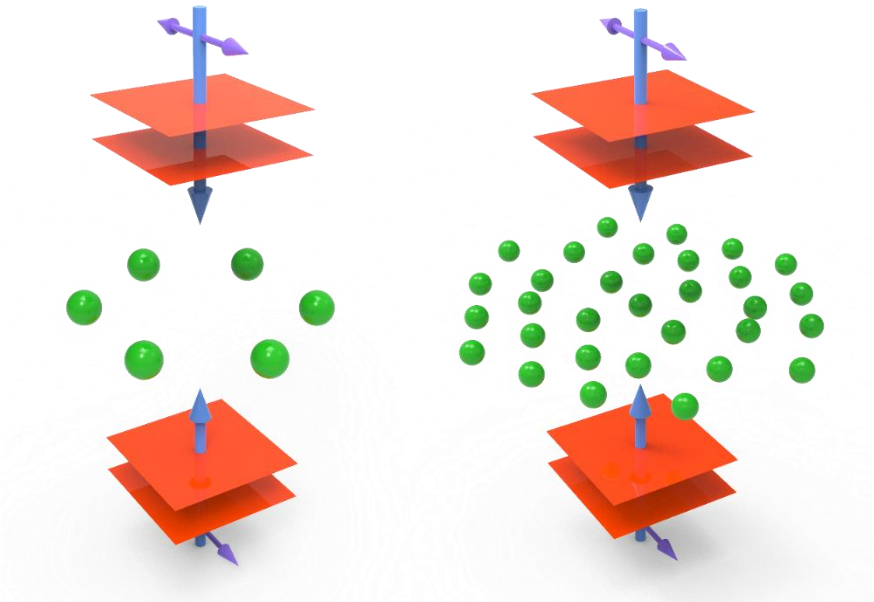

Supplementary Fig. 13| **Planar optically bound clusters.** The number of particles considered in the main text varies from $N = 2$ to $N = 55$. As shown in Fig. S1.3, for the same number of particles, there can be multiple stable configurations. Two examples of planar optically-bound structures are shown, and the remainders are not shown.

Supplementary Note 4: Optical Binding Simulations with Hydrodynamic Interaction

It is expected that sufficiently strong damping can remove the excess energy of the complex modes pumped in by light, as discussed in the main text, preventing energy accumulation, and rendering the cluster stable. We now consider the stability of a collection of particles under optical forces, damping forces, and hydrodynamic coupling [1]. As expected, the introduction of strong ambient damping stabilizes the cluster with complex modes.

For a medium with heavy damping, such as water, the inertial term can be neglected such that the optical forces are balanced by the Stokes drag. The velocity of the ith particle is given by

$$\mathbf{V}_{\text{i}} = \sum_{\text{j}=1}^{N} \left[\frac{\overleftrightarrow{\mathbf{I}}}{\gamma} \delta_{\text{ij}} + \frac{3r_{\text{S}}}{4\gamma r_{\text{ij}}} (1-\delta_{\text{ij}}) \left(\overleftrightarrow{\mathbf{I}} + \frac{\mathbf{r}_{\text{ij}}\mathbf{r}_{\text{ij}}}{r_{\text{ij}}^2} \right) \right] \mathbf{F}_{\text{j}}, \tag{S4.1}$$

where the hydrodynamic coupling is governed by the Oseen tensor [6], $\gamma = 6\pi\eta r_{\text{S}}$ is the friction coefficient with viscosity $\eta = 0.89\times10^{-3}\,\text{Pa}\cdot\text{s}$ in water, δ_{ij} denotes the Kronecker delta function, $\mathbf{r}_{\text{ij}}$ is the vector pointing from particle *i* to *j*, $\overleftrightarrow{\mathbf{I}}$ is the unit tensor, and $\mathbf{F}_{\text{j}}$ denotes the optical force exerted on the particle *j*. We take the linear approximation for the optical force: $\mathbf{F} = \overleftrightarrow{\mathbf{K}} \cdot \Delta\mathbf{X}$, with $\Delta\mathbf{X}$ denoting the displacement away from the equilibrium position.

Consider particles immersed in a triangular optical lattice, as shown in the first column of Supplementary Fig. 14. Such structures possess multiple pairs of complex modes, and one may simulate their trajectories using the Verlet algorithm. Consider particles in vacuum (see the second column of Supplementary Fig. 14), which experience only optical forces. The particles initially deviate from the hotspots of the lattice by only a few nanometers, but the cluster vibrates with increasing amplitude due to the optical forces associated the complex modes. As a result, the cluster will eventually disintegrate. For particles in water (see the third column of Supplementary Fig. 14), at the laser intensity under consideration, one has to consider only optical and hydrodynamic forces [1]. Although the particles are initially given a random deviation from the hotspots by $\sim 0.1\lambda$, their energy is quickly dissipated by the damping effect of the water, and the particles return to their hotspots.

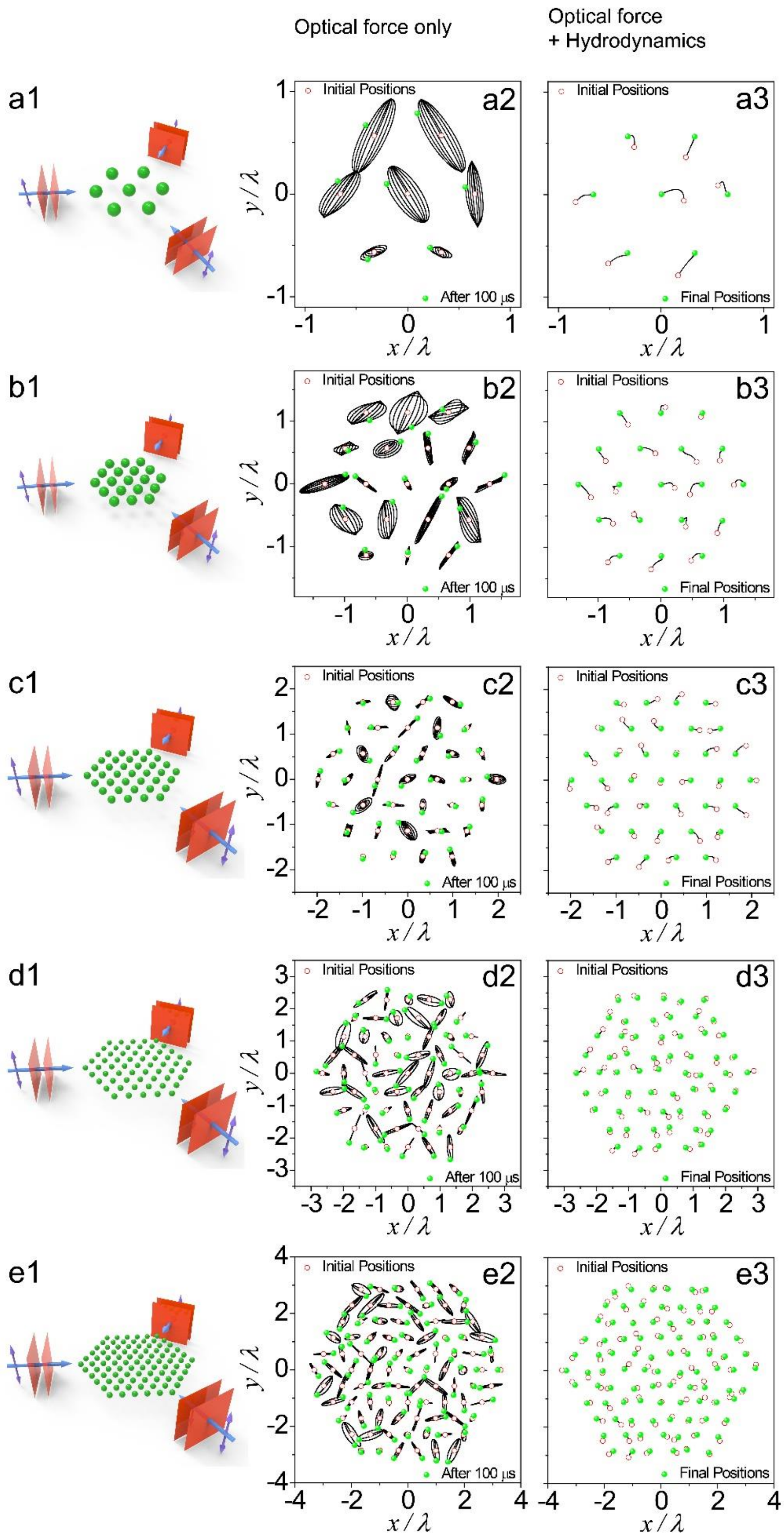

Supplementary Fig. 14| **Particles trajectories for triangular lattices with different sizes 7 (a), 19 (b), 37 (c), 61 (d), 97 (e).** 2nd column: optical force only, the particles vibrate with imcreasing amplitudes and eventually disintegrate. 3rd column: optical force + hydrodynamic forces, the particles return to the equilibrium positions even if they are perturbed by relatively large initial deviations from the equilibrium. The relative refractive index and the particle radius is set as $n_r = 1.1$ and $r_S = 0.2\lambda$, respectively.

Supplementary Note 5: Non-Hermitian Theory for Transverse and Longitudinal Optical Binding in Vacuum

While the interaction between optically bound particles is nonconservative thus Hermitian, small and highly symmetric systems may be treated as approximately Hermitian. Here, we consider two examples, namely longitudinal [Svak et al., Optica 8, 220-229 (2021)] and transverse [Arita et al., Optica 5, 910-917 (2018)] optical binding in vacuum. We find that the Hermitian treatment adopted in those experiments are indeed a good approximation that captures the essence of the interaction. However, more general non-Hermitian treatment shows that strictly speaking, these systems are non-Hermitian in nature. Non-Hermiticity becomes important when more particles are involved or when spatial symmetry is broken, echoing the main conclusion in the main text. This strongly supports the need of a non-Hermitian theory for optical binding and our non-Hermitian approach yields interesting and timely new insight.

A. Longitudinal optical binding in counter-propagating Gaussian beams [for configurations discussed in Svak et al., Optica 8, 220-229 (2021)].

Supplementary Figure 15(a) and (b) show the longitudinal binding of 2 and 3 spheres using counter-propagating Gaussian beam in vacuum (or very low air pressure), as discussed in Ref. [7]. In our numerical simulations, the N particles are initially separated by λ. Equilibrium positions are then found by using molecular dynamics simulations.

For 2 silica spheres (Supplementary Fig. 15(a)) with $r_S = 0.2\mu\text{m}$ and $n_r = 1.45$, the 3D force matrix is

$$\overleftrightarrow{\mathbf{K}}_{3D}^{2\text{Spheres}} = \begin{bmatrix} -2.59810 & 0 & 0 & 0.11257 & 0 & 0 \\ 0 & -1.99241 & 0 & 0 & 0.11248 & 0 \\ 0 & 0 & -8.31732 & 0 & 0 & -0.01410 \\ 0.11257 & 0 & 0 & -2.59810 & 0 & 0 \\ 0 & 0.11248 & 0 & 0 & -1.99241 & 0 \\ 0 & 0 & -0.01410 & 0 & 0 & -8.31732 \end{bmatrix} \frac{\text{pN}}{\mu\text{m}}, \qquad \text{(S5.1)}$$

which is symmetric (Hermitian). Its Hermiticity is protected by reflection symmetries, as explained in the Supplementary Note 2.

However, for $N = 3$ (Supplementary Fig. 15(b)), the force matrix becomes asymmetric

$$\overleftrightarrow{\mathbf{K}}_{\text{3D}}^{\text{3Spheres}} =$$

$$\begin{bmatrix} -0.42707 & 0 & 0 & 0.04942 & 0 & 0 & 0.012854 & 0 & 0 \\ 0 & -0.41639 & 0 & 0 & 0.06014 & 0 & 0 & 0.01384 & 0 \\ 0 & 0 & -4.56961 & 0 & 0 & 0.13661 & 0 & 0 & 0.07079 \\ 0.04578 & 0 & 0 & -0.49791 & 0 & 0 & 0.04578 & 0 & 0 \\ 0 & 0.05737 & 0 & 0 & -0.49882 & 0 & 0 & 0.05737 & 0 \\ 0 & 0 & 0.15475 & 0 & 0 & -5.08720 & 0 & 0 & 0.15475 \\ 0.01285 & 0 & 0 & 0.04942 & 0 & 0 & -0.42707 & 0 & 0 \\ 0 & 0.01384 & 0 & 0 & 0.06015 & 0 & 0 & -0.41639 & 0 \\ 0 & 0 & 0.07079 & 0 & 0 & 0.13661 & 0 & 0 & -4.56961 \end{bmatrix} \frac{\text{pN}}{\mu\text{m}}$$

(S5.2)

With increasing degrees of freedom, the available symmetries are not sufficient to keep the matrix Hermitian. It is noteworthy that in the geometry of Supplementary Fig.15, *x*, *y*, and *z* directions are decoupled. Accordingly, we can decompose $\overleftrightarrow{\mathbf{K}}_{\text{3D}}^{\text{3Spheres}}$ into three asymmetric matrices:

$$\overleftrightarrow{\mathbf{K}}_{\text{1D,x}}^{\text{3Spheres}} = \begin{bmatrix} -0.42707 & 0.04942 & 0.012854 \\ 0.04578 & -0.49791 & 0.04578 \\ 0.01285 & 0.04942 & -0.42707 \end{bmatrix} \frac{\text{pN}}{\mu\text{m}}, \tag{S5.3}$$

$$\overleftrightarrow{\mathbf{K}}_{\text{1D,y}}^{\text{3Spheres}} = \begin{bmatrix} -0.41639 & 0.06014 & 0.01384 \\ 0.05737 & -0.49882 & 0.05737 \\ 0.01384 & 0.06015 & -0.41639 \end{bmatrix} \frac{\text{pN}}{\mu\text{m}}, \tag{S5.4}$$

and

$$\overleftrightarrow{\mathbf{K}}_{\text{1D,z}}^{\text{3Spheres}} = \begin{bmatrix} -4.56961 & 0.136609 & 0.07079 \\ 0.15475 & -5.08720 & 0.15475 \\ 0.07079 & 0.13669 & -4.56961 \end{bmatrix} \frac{\text{pN}}{\mu\text{m}}. \tag{S5.5}$$

Nevertheless, the non-Hermiticity is weak such that their eigenvalues are essentially real, signifying stable optical binding. Thus it is a good approximation to adopt the Hermitian theory as in Ref. [7]. Sphere chains with 4-10 spheres are also considered, and their eigenvalues are exactly real.

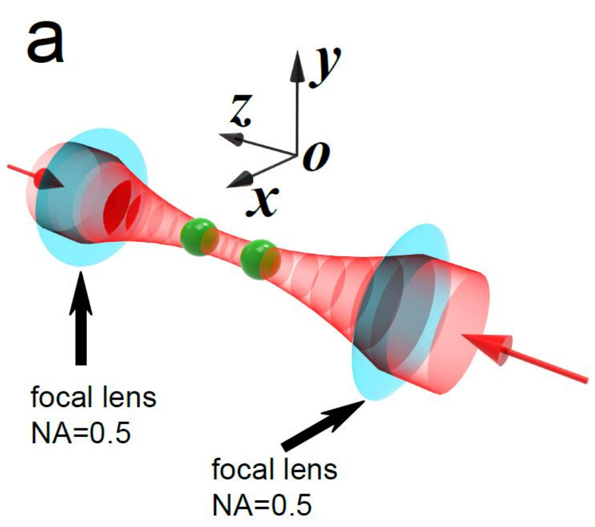

Supplementary Fig. 15| **Longitudinal optical binding of *N* spheres by counter propagating focused beam in vacuum.** The numerical apertures of the two lenses (light blue) are 0.5. The incident beams are linearly polarized with a wavelength of $\lambda = 1.064$ μm. Different particle radius, particle composition, and particle numbers are considered. The beam power for each is normalized to 1.0 mW. (a) $N = 2$. (b) $N = 3$. We also considered other particle numbers with $N = 4 - 10$, but no complex eigenvalue (vibration frequency) is found.

Nonetheless, strictly speaking, the system is non-Hermitian, and the Hermitian approximation is not always valid. For example, we deliberately break the mirror symmetry along *z*-axis by assigning a different numerical apertures (0.5 and 0.6) to the two lenses. Under such condition, the non-Hermitian (antisymmetric) components will be enhanced. This can be most easily seen from the fact that the scattered light from the right sphere to the left sphere is now different from that of the left to right, and therefore the two matrix elements (force on right sphere due to the left and force on left sphere due to right) will be different, therefore an antisymmetric component has now emerged. We calculated the force matrices for 4 silica spheres illustrated in Supplementary Fig. 16, with radius ranging from 0.20 μm to 0.28 μm. The real and imaginary parts of the eigenvalues for force matrix $\overleftrightarrow{\mathbf{K}}_{\mathrm{1D,z}}^{\mathrm{4Spheres}}$ versus the particle radius are plotted in Supplementary Fig. 16 (b) and (c), respectively. Complex eigenvalues have emerged. The minimally required viscosity for stably

binding the particles is also plotted in Supplementary Fig. 16(d). The beam power for each incident beam is normalized to 1.0 mW for the data shown in Supplementary Fig. 16. According to the definition of $\eta^{\mathrm{i}}_{\mathrm{Critical}} = \frac{\gamma^{\mathrm{i}}_{\mathrm{Critical}}}{6\pi r_{\mathrm{S}}} = \frac{\sqrt{m}|\mathrm{Im}[K_{\mathrm{i}}]|/\sqrt{|\mathrm{Re}[K_{\mathrm{i}}]|}}{6\pi r_{\mathrm{S}}}$, the critical viscosity will be $\eta^{\mathrm{i}}_{\mathrm{Critical}} = \frac{\gamma^{\mathrm{i}}_{\mathrm{Critical}}}{6\pi r_{\mathrm{S}}} = \frac{\sqrt{m}|\mathrm{Im}[K_{\mathrm{i}}]|/\sqrt{|\mathrm{Re}[K_{\mathrm{i}}]|}}{6\pi r_{\mathrm{S}}}\sqrt{P_0}$, where the power for a single incident beam is P_0 mW.

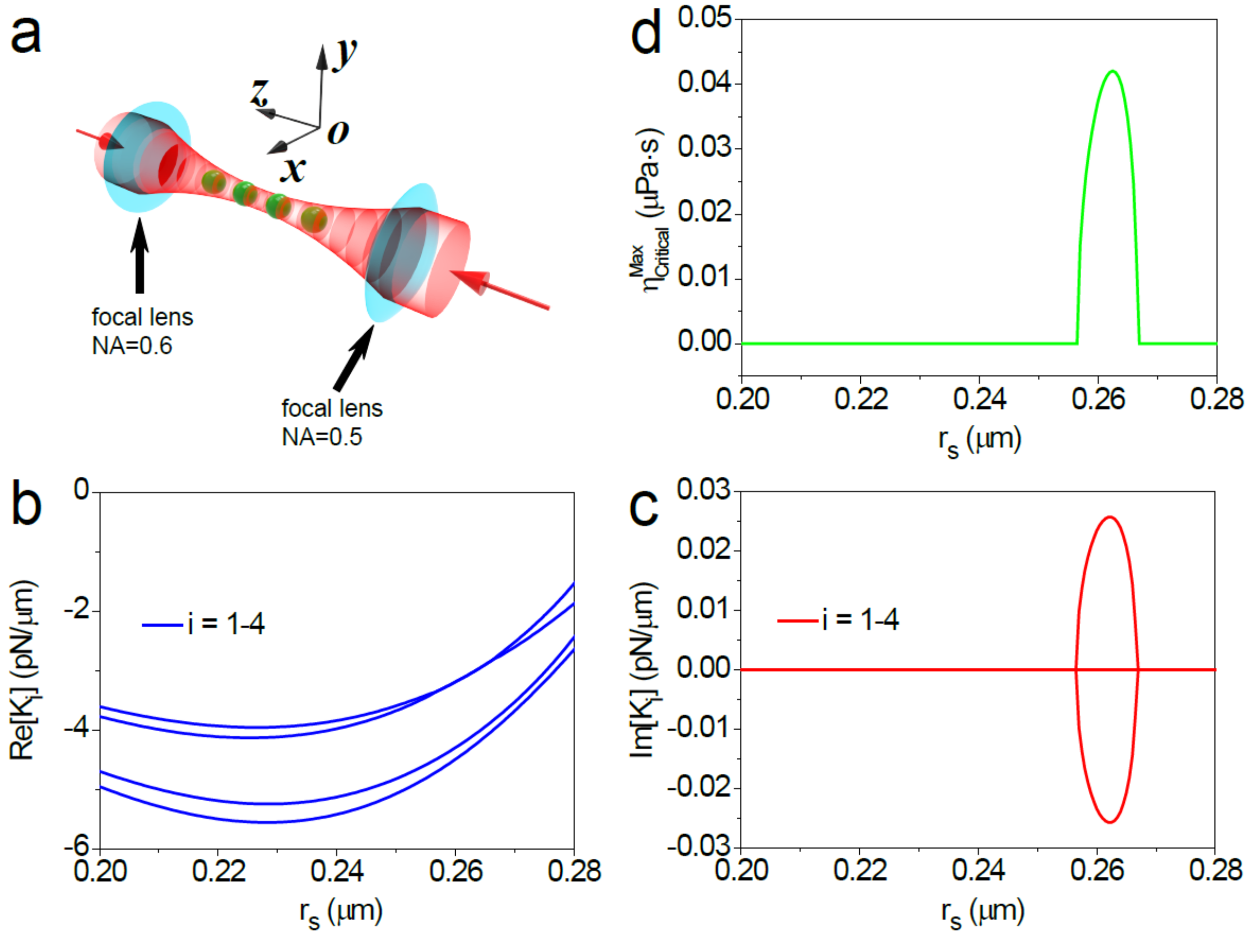

Supplementary Fig. 16| **Optical binding of 4 spheres using counter propagating focused beam in vacuum.** The numerical apertures of the focusing lenses (light blue) are 0.6 (left beam) and 0.5 (right beam), which breaks the mirror symmetry on the *xy*-plane. For silica spheres with $\mathrm{n_r} = 1.45$, the real and imaginary parts of the eigenvalues for force matrix $\overleftrightarrow{\mathbf{K}}^{\mathrm{4Spheres}}_{\mathrm{1D,z}}$ are plotted in (b) and (c), respectively. (d) The minimal requirement for the background viscosity to stabilize the cluster. The density of the particle is set as 2.650 g/cm^3. The beam power for each is normalized to 1.0 mW, and $\lambda = 1.064$ μm.

B. Lateral optical binding for two particles in vacuum

Lateral (or transverse) optical binding in vacuum has been investigated experimentally by Ref. [8] for a pair of vaterite microparticles levitated, bounded, and spined by a pair of parallel circularly polarized Gaussian beams. It is an important example in vacuum binding. However, simulation of the binding of the anisotropic vaterite particles is beyond the scope of this work. Instead, we consider a pair of homogeneous particles. The levitated particles will be close to (away from) the focus if the NA of the focusing lenses is large (small) or when the particle is heavy (light). Our simulations show that for particles levitated close to the focus, the eigenvalues are always complex, implying instability in the absence of sufficient damping, while for particles away from the focus, the stability along the x and y directions is not guaranteed. We stress that such instability may not occur for spinning particle, as spinning can stabilize the trapping, as stated in Ref. [8].

Accordingly, we consider the levitation and binding of two homogeneous dielectric spheres in the lateral optical binding settings. Lateral optical binding refers to the geometry depicted in Supplementary Fig. 17(a), where the two 2.2 μm radius particles (green spheres) are separated by 10 μm. The physical ground for the particle-particle coupling can be envisioned as originating from the multiple scattering between the two particles. To be specific, the scattered light from one sphere reaches the other one, which modifies the intensity distribution near the second sphere, and thus exerts a force on it, resulting in the mechanical coupling. Owing to the large refractive index contrast and lack of damping, vacuum trapping is typically difficult. Instead, the particles can be levitated, where the equilibrium is established by balancing the upward radiation pressure with the particle weight. Such stable equilibrium is found at $z \approx 17$ μm (assuming a mass density of 2.710 g/cm^3 for the particle), marked by the intersection point of the redlines in Supplementary Fig. 17(e).

In Supplementary Fig. 17(b-c), we tune the particle's mass density such that its equilibrium varies from $z = 0$ to 40 μm, and plot the real and imaginary parts of the eigenvalues for the $\overleftrightarrow{\mathbf{K}}$. Here, we only show the complex eigenmodes (K_i), with $i = 1 - 4$. Clearly, complex eigenvalues are observed when the particles are near the focal points $z = 0$ μm. The minimum ambient damping required to stabilize the particles is plotted in Supplementary Fig. 17(d).

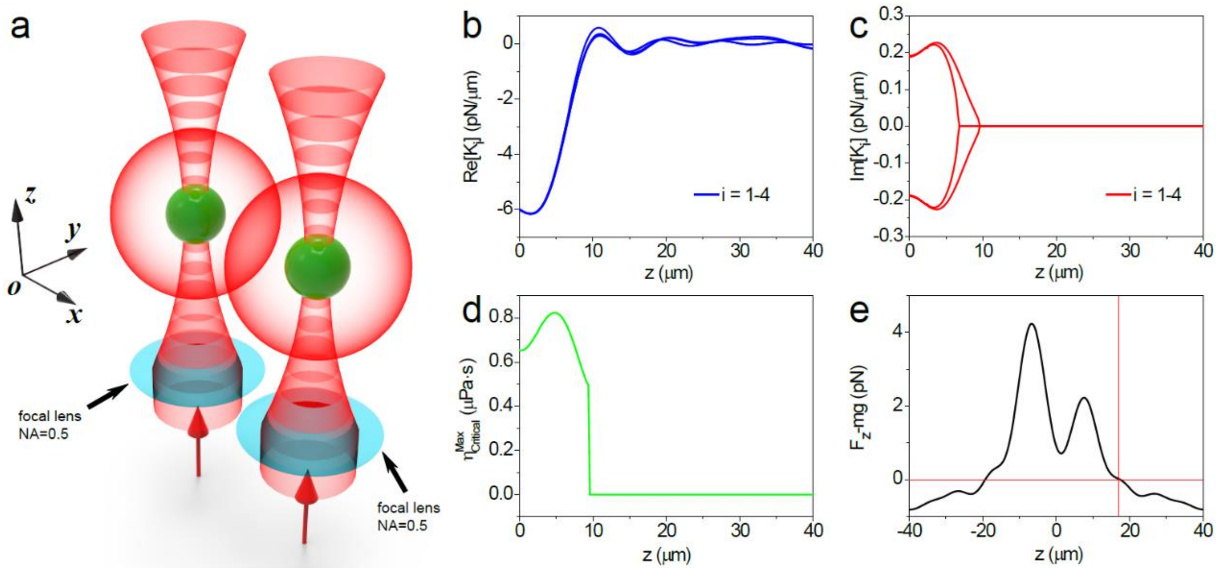

Supplementary Fig. 17| **Lateral optical binding of two dielectric spheres ($n_r = 1.57$) levitated in vacuum by two focused circularly polarized Gaussian beam.** (a) Schematic plot for the geometry. The incident beams are focused by the focal lenses (cyan circular plates) with the same numerical aperture 0.5, and the wavelength is 1.064 μm. The two spheres are trapped at the same height (b-c) Real (b) and imaginary (c) parts of the eigenvalues for the force matrix $\overleftrightarrow{\mathbf{K}}$ versus the particle position (z). (d) The critical viscosity (minimal requirement for stable trapping and binding) versus the particle position (z). (e) Difference between the optical force along z-direction and the gravity of one particle versus the particle positions (z). The intersection point of the red lines marks the equilibrium position when the mass density of the particle is set to 2.710 g/cm^3. The beam power for each beam is 7.0 mW and $\lambda = 1.064$ μm.

References

1 Gordon, R., Kawano, M. & Blakely, J. T. Optohydrodynamic theory of particles in a dual-beam optical trap. *Phys. Rev. B* **77**, 245125 (2008).

2 Ng, J. Lin, Z. F., Chan, C. T. & Sheng, P. Photonic clusters formed by dielectric microspheres: Numerical simulations. *Phys. Rev. B* **72**, 085130 (2005).

3 Seyranian, A. P. & Mailybaev, A. A. Multiparameter stability theory with mechanical applications. *World Scientific* (2003).

4 Ng, J. Light-induced forces on small particles. Hong Kong University of Science and Technology (Hong Kong) (2005).

5 Burns, M. M., Fournier, J.-M. & Golovchenko, J. A. Optical matter: crystallization and binding in intense optical fields. *Science* **249**, 749 (1990).

6 Meiners, J. C. & Quake, S. R. Direct measurement of hydrodynamic cross correlations between two particles in an external potential. *Phys. Rev. Lett.* **82**, 2211 (1999).

7 Svak, V., Flajšmanová, J., Chvátal, L., Šiler, M., Jonáš, A., Ježek, J., Simpson, S. H., Zemánek, P. & Brzobohatý, O. Stochastic dynamics of optically bound matter levitated in vacuum. *Optica* **8**, 220-229 (2021).

8 Arita, Y., Wright, E. M. & Dholakia, K. Optical binding of two cooled micro-gyroscopes levitated in vacuum. *Optica* **5**, 910-917 (2018).
