# Supplementary material for: Non-Hermitian physics for optical manipulation uncovers inherent instability of large clusters
Source: Nat Commun. 2021 Nov 15;12:6597. doi: 10.1038/s41467-021-26732-8 (PMC8593170; doi:10.1038/s41467-021-26732-8)
Supplement: Supplementary file 2 — Description of Additional Supplementary Files [file 41467_2021_26732_MOESM2_ESM.pdf]

Description of Additional Supplementary Files

Supplementary Movie 1: Trajectories of optically bound particles in a triangular optical lattice in vacuum.

Supplementary Movie 2: Trajectories of optically bound particles in a triangular optical lattice in water.
